# Longitudinal Meta-cohort study protocol using systems biology to identify vaccine safety biomarkers

**DOI:** 10.1016/j.vaccine.2025.127504

**Published:** 2025-07-26

**Authors:** Joann Diray-Arce, Ana C. Chang, Sara Moradipoor, Donato Amodio, Bruce Carleton, Wan-Chun Chang, Nigel W. Crawford, Meagan Karoly, Annmarie Hoch, Kerry McEnaney, Tahir S. Kafil, Mahitha Donthireddy, Sarah K. Steltz, Simon D. van Haren, Asimenia Angelidou, Kinga K. Smolen, Hanno Steen, Jessica Lasky-Su, Huyen Tran, Peter Liu, C. Buddy Creech, Clare L. Cutland, Helen Petousis-Harris, Ishac Nazy, Rae S.M. Yeung, Sonali Kochhar, Steve Black, Nicholas Wood, Dale Nordenberg, Paolo Palma, Inna G. Ovsyannikova, Richard B. Kennedy, Gregory A. Poland, Al Ozonoff, Robert T. Chen, Ofer Levy, Karina A. Top, International Network of Special Immunization Services (INSIS) Members

**Affiliations:** a Precision Vaccines Program, Department of Pediatrics, Boston Children’s Hospital, Boston, MA 02115, USA; b Harvard Medical School, Boston, MA 02115, USA; c Department of Pediatrics, University of Alberta, Edmonton, AB T6G 1C9, Canada; d Clinical and Research Unit of Clinical Immunology and Vaccinology, Bambino Gesù Children’s Hospital, IRCCS, Rome 00165, Italy; e Department of Systems Medicine, University of Rome Tor Vergata, Rome 00133, Italy; f Department of Pediatrics and BC Children’s Hospital Research Institute, University of British Columbia, Vancouver, BC V5Z 4H4, Canada; g Murdoch Children’s Research Institute (MCRI_AEFI-CAN) & University of Melbourne, Parkville, VIC 3052, Australia; h Department of Cardiovascular Medicine, Mayo Clinic, Rochester, MN 55905, USA; i Department of Neonatology, Beth Israel Deaconess Medical Center, Boston, MA 02215, USA; j Department of Pathology, Boston Children’s Hospital, Boston, MA 02115, USA; k Channing Division of Medicine, Brigham and Women’s Hospital, Boston, MA 02115, USA; l Monash University, Melbourne, VIC 3004, Australia; m University of Ottawa Heart Institute, Ottawa, ON K1W 4W7, Canada; n Vaccine Research Program, Vanderbilt University Medical Center, Nashville, TN 37232, USA; o African Leadership in Vaccinology Expertise (Wits-Alive), Faculty of Health Sciences, University of the Witwatersrand, Parktown, Johannesburg 2050, South Africa; p Global Vaccine Data Network and School of Population Health, University of Auckland, 1142, New Zealand; q Department of Medicine McMaster University, Hamilton, ON L8S 4K1, Canada; r Michael G. DeGroote Centre for Transfusion Research, McMaster University, Hamilton, Ontario L8S 4K1, Canada; s Department of Biochemistry and Biomedical Sciences, McMaster University, Hamilton, Ontario L8S 4K1, Canada; t Cell and Systems Biology Research Program, The Hospital for Sick Children, Departments of Paediatrics, Immunology and Institute of Medical Sciences, University of Toronto, Toronto, ON M5G 1X8, Canada; u Global Healthcare Consulting, New Delhi 110024, India; v University of Washington, Seattle, WA 98915, USA; w University of Sydney, Sydney, NSW 2006, Australia; x Thrüve, Bronx, NY, USA; y Vaccine Research Group, Division of General Internal Medicine, Mayo Clinic, Rochester, MN 55905, USA; z Broad Institute of MIT and Harvard, Cambridge, MA 02142, USA; aa Safety Platform for Emergency vACcines (SPEAC), Brighton Collaboration, The Task Force for Global Health, Decatur, GA 30030, USA; ab Brighton Collaboration, Task Force for Global Health, Decatur, GA 30030, USA; ac Li Ka Shing Institute of Virology and Women’s and Children’s Health Research Institute, University of Alberta, Edmonton, AB T6G 2E1, Canada; ad Departments of Community Health & Epidemiology, Dalhousie University, Halifax, NS B3H 4R2, Canada

**Keywords:** Adverse event of following immunization, AEFI, Adversomics, COVID-19 vaccines, Myocarditis, Pericarditis, VITT, TTS, PF4, Vaccine safety, Systems biology, Multi-omics, Biomarker discovery, INSIS

## Abstract

The International Network of Special Immunization Services (INSIS) was established to investigate the causes and risk factors of rare adverse events following immunizations (AEFIs) and develop immunization strategies for mitigating or preventing risk for individuals with prior AEFIs or at risk of AEFIs. INSIS integrates clinical data with multi-omic technologies (e.g., transcriptomics, proteomics, metabolomics) through a global consortium of clinical networks, leading immunology, pharmacogenomics teams to uncover the molecular mechanisms behind AEFIs. The network ensures accurate and standardized data collection and analysis through rigorous data management and quality assurance processes. INSIS also implements harmonized case definitions and protocols for collecting data and samples related to rare AEFIs, such as myocarditis, pericarditis, and Vaccine-Induced Immune Thrombocytopenia and Thrombosis (VITT) after COVID-19 vaccinations. This protocol outlines the comprehensive approach to enhance risk-benefit assessments of vaccines across populations, identify actionable biomarkers to inform discovery and development of safe vaccines, and support personalized vaccination strategies.

## 1. Introduction

Vaccines are among the most effective public health interventions, playing a critical role in preventing and controlling multiple public health emergencies and pandemics, such as influenza A/H1N1 in 2009, the Ebola outbreak in West Africa in 2014, COVID-19, and most recently monkey pox (mpox). Advances in biotechnology have allowed for the licensure of many new vaccines. However, similar to other medical interventions, they have potential risks. While serious adverse events following immunizations (AEFI)’s were recognized after introduction of smallpox and diphtheria antitoxin vaccinations in 18th and 19th century, respectively, the first scientific review on Hazards of Immunization, summarizing mostly case reports and case series, was only published in 1967 [1].

While much progress has been made since the 1990’s, especially in high income countries, in early detection and quantification of vaccine risks using both passive and active surveillance for hypothesis generation and testing of AEFI’s [2], there has been limited progress in understanding their biological and molecular mechanisms to mitigate or prevent newly detected vaccine risk [3]. The rarity of these serious vaccine risks, the relative immaturity of the underlying systems biology science, plus limited funding contributed to this limited progress.

COVID-19 vaccines were developed at unprecedented speed, receiving authorization under accelerated timelines and procedures within 10 months of the pandemic being declared [4,5]. These vaccines have been credited with preventing up to 20 million deaths from COVID-19 in the first year after their roll-out [6]. While the overwhelming benefits of COVID-19 vaccination continue to outweigh risks [7], rare AEFIs were identified through post-market surveillance [8]; two of which, Thrombosis with Thrombocytopenia Syndrome (TTS), including Vaccine-Induced Immune Thrombocytopenia and Thrombosis (VITT) and myocarditis/pericarditis were most prominent. Additionally, increased risks of immune thrombocytopenic purpura (ITP) and Guillain-Barré Syndrome (GBS) have also been observed with adenoviral vector vaccines [9,10]. Given the ongoing use of adenoviral vector platforms in developing vaccines against emerging diseases, understanding the pathophysiology of VITT and other AEFIs associated with these vaccines remains a priority.

VITT has been linked to adenoviral vector COVID-19 vaccines (e.g., ChAdOx1 and Ad.26.COV-2.S). First reported in March 2021, VITT is characterized by rapidly progressive thrombosis, particularly in cerebral venous sinuses and splanchnic circulation veins, accompanied by thrombocytopenia and markedly elevated D-dimer and anti-platelet factor 4 (PF4) antibodies driving platelet activation [11-15]. The incidence of VITT ranges from 0.1 to 1.2 cases per 100,000 vaccinations following Ad26.COV-2.S and 1 to 5 per 100,000 following the first dose of ChAdOx1 vaccine [16]. Early diagnosis and treatment have reduced case fatality rates from as high as 40 % to 13–15 % [17].

Myocarditis and pericarditis emerged as safety signals following the rollout of COVID-19 mRNA vaccines. Although these vaccines have been pivotal in controlling the pandemic, there is an increased incidence of myocarditis and pericarditis, particularly among young males aged 12–29 years after the second dose [18,19-25]. Initial data suggested most patients appeared to recover quickly. Ongoing studies are focused on determining long-term outcomes, including resolution of cardiac MRI changes and cardiac-specific biomarkers, with some studies reporting persistence of MRI changes and symptoms in a subset of patients [26]. Importantly, despite these rare events, the benefits of COVID-19 vaccination in preventing severe disease, hospitalization, and death continue to far outweigh the risks. The mRNA platform is now being used to develop vaccines against a range of other diseases including influenza, Respiratory Syncytial Virus (RSV), Nipah, and Lassa fever, as well as for non-infectious indications such as cancer [11,27].

Myocarditis and pericarditis have also been reported following non-mRNA COVID-19 vaccines, including the adjuvanted protein subunit vaccine Novavax NVX-CoV2373 and AstraZeneca’s ChAdOx1 [23]. Early studies suggest that the spike protein may contribute to these rare cardiac events [28,29]. Ongoing research into the underlying biological mechanisms is essential to refine our understanding of vaccine safety. Continued surveillance and investigation are critical to ensuring public confidence and the safety of vaccination programs.

The International Network of Special Immunization Services (INSIS) was formed in 2020 to develop a “first-of-its kind” global network for investigating rare AEFIs, starting with AEFIs associated with COVID-19 vaccination [30]. When TTS/VITT and myocarditis emerged as vaccine safety signals in 2021, specialist clinical networks were engaged to develop guidance for clinical investigation and management [31]. In some cases, these networks were able to quickly collect clinical data and biosamples from patients in their care in real-time. Clinical immunization assessment networks, such as the Canadian Special Immunization Clinic (SIC) Network and Australian Adverse Event Following Immunization-Clinical Assessment Network (AEFI-CAN), mobilized to support these efforts and evaluate patients requiring additional COVID-19 vaccine doses to complete the series. The Global Vaccine Data Network (GVDN) also initiated a study of genetic markers of COVID-19 vaccine adverse events of special interest (AESI), with samples collected by several INSIS sites, including those associated with SIC Network and AEFI-CAN [32].

INSIS has collaborated with these networks to harmonize data and sample collection to conduct downstream systems biology analyses and support genomics studies via GVDN. This collaboration ensures a sufficient sample size for robust analyses, supports multi-omics analysis of the collected samples, and enhances inter-operability of data to enable larger and more impactful analyses, while expanding the potential for discovery and replication.

INSIS combines clinical investigation with immunologic studies and systems biology “adversomics” such as transcriptomics, proteomics, epigenetics, and metabolomics. Adversomics describes the use of omics and other technologies in studying adverse reactions related to vaccines. These technologies measure relevant categories of molecules (e.g., DNA, RNAs, proteins, metabolites) in a given sample investigating possible patterns related to vaccine recipients with adverse events versus those without such adverse events [33,34]. This comprehensive approach has the significant advantage of not making any assumptions regarding potentially relevant molecular mechanisms but rather casting a broad net to define molecular pathways associated with, and potentially contributing to, AEFIs.

Studying AEFIs is crucial because they negatively impact the health of those experiencing them as well as vaccine uptake and public confidence in vaccination programs [35]. Understanding the clinical spectrum, risk factors, and underlying mechanisms of AEFIs informs the risk-benefit assessment of vaccines [36]. Additionally, these insights are essential for the discovery and development of safe vaccines, particularly as new platforms are employed to protect against emerging diseases. Together, these approaches will enable more informed decision-making, ultimately contributing to better public health outcomes. The infrastructure and methodologies developed by INSIS can be leveraged for future studies of other AEFIs for different vaccines against current and emerging pathogens or novel vaccines for non-infectious indications (e.g., vaccines against cancer, allergy or drug overdose).

In this report, we describe INSIS’ current approach and methodology, including the recruitment of participants from multiple global sites, harmonization of data collection, and use of advanced laboratory techniques to analyze biological samples. This also highlights the importance of understanding AEFIs to inform vaccine safety, improve public confidence, and support the development of personalized vaccination strategies.

## 2. Materials and methods

**Fig. 1.**
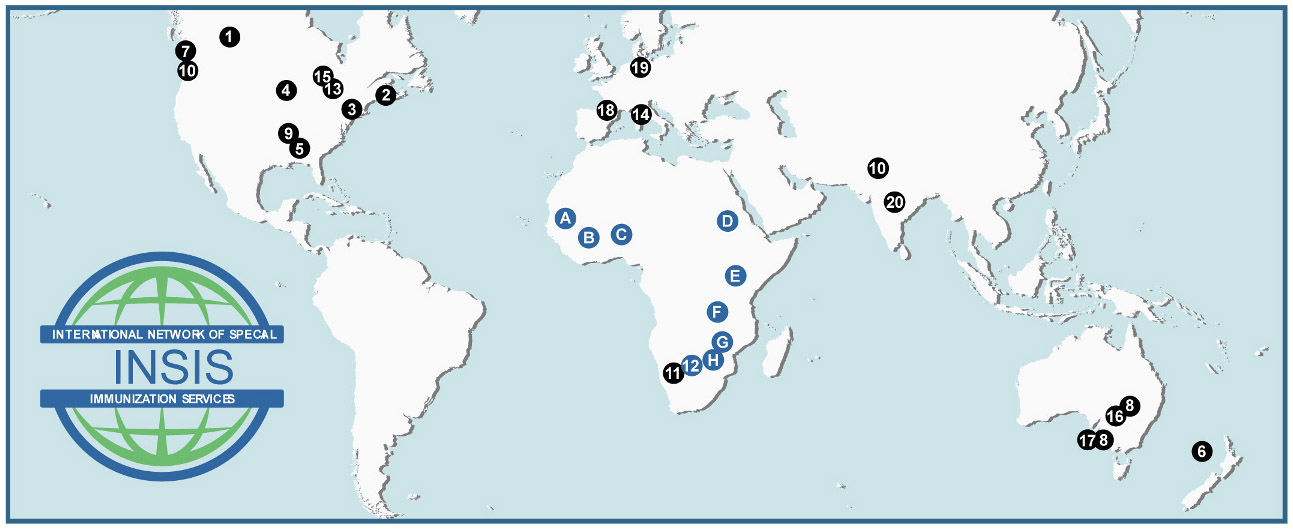
Map of International Network of Special Immunization Services formal collaborating partners. The participating sites are as follows: ^1^INSIS Management Office at the University of Alberta, Edmonton, Alberta, Canada; ^2^The Hospital for Sick Children (SickKids), Toronto, Ontario, Canada; ^3^Precision Vaccines Program at Boston Children’s Hospital, Boston, Massachusetts, United States; ^4^Mayo Vaccine Research Group, Rochester, Minnesota, United States; ^5^Brighton Collaboration, Decatur, Georgia, United States; ^6^Global Vaccine Data Network, Auckland, New Zealand; ^7^Canadian Pharmacogenomics Network for Drug Safety, Vancouver, British Columbia, Canada; ^8^Murdoch Children’s Research Institute, Melbourne, Australia; ^9^Vanderbilt Vaccine Research Program, Nashville, Tennessee, United States; ^10^University of Washington, Seattle, Washington, United States; ^11^University of the Witwatersrand, Johannesburg, South Africa; ^12^ALIVE Network (African Leadership in Vaccinology Expertise), Johannesburg, South Africa; ^13^McMaster University, Hamilton, Ontario, Canada; ^14^Ospedale Pediatrico Bambino Gesù, Rome, Italy; ^15^University of Ottawa Heart Institute, Ottawa, Ontario, Canada; ^16^University of Sydney, Sydney, Australia; ^17^Monash University, Clayton, Australia; ^18^VAC4EU, Brussels, Belgium; ^19^University of Southern Denmark; Denmark; ^20^Global Healthcare Consulting, New Delhi, India; A. Sites in Bamako, Mali; B. Navrongo Health Research Centre, Navrongo, Ghana; C. National site in Nigeria; D. Site in Gondar, Ethiopia; E. Site in Kilifi, Kenya; F. National site in Malawi; G. Maputo City, Mozambique; H. National site in Eswatini.

This multi-center, multi-national observational case-control study aims to investigate the pathophysiology of TTS/VITT, myocarditis, and pericarditis following COVID-19 vaccination. Participants are being recruited from INSIS partner sites (Fig. 1).

### 2.1. Study procedures

INSIS partner sites providing retrospective data identified eligible participants through various means, including clinical referrals, samples submitted for laboratory testing (e.g., PF4 antibody testing for COVID-19 vaccine-associated VITT), and passive and active AEFI surveillance. Eligible participants underwent clinical assessments to confirm the diagnosis and rule out other causes of myocarditis, pericarditis, and TTS/VITT, using diagnostic imaging and other methods as per routine clinical care. Investigations and additional referrals were completed as indicated, according to local and national policies and procedures and physician discretion. INSIS investigators harmonized clinical assessments to the extent possible.

### 2.2. Ethics statement

All procedures will be performed in compliance with relevant laws and institutional guidelines as approved by the appropriate institutional committees. Informed consent will be obtained prior to study procedures.

### 2.3. Clinical data collection

All INSIS sites will transfer the raw data from their database to the INSIS REDCap database, which is designed to capture general common data elements for all participants including age, country of enrollment, biological sex, self-reported gender, COVID-19 vaccination details, relevant past medical history, relevant medications, and details of the AEFI/condition including interval from vaccination to symptom onset (if applicable), specific symptoms, and diagnostic results. Details of the AEFIs will be captured in forms derived from the Brighton Collaboration Case Definitions for TTS/VITT and myocarditis/pericarditis to ensure each case is evaluated using consistent criteria. At each follow-up visit, data will be collected on any changed signs or symptoms including those associated with myocarditis, pericarditis, or TTS/VITT to detect persistent or recurrent disease activity.

### 2.4. Case definitions

#### 2.4.1. Myocarditis/pericarditis

The Brighton Collaboration case definitions ([37], [38])will be used for both post-vaccine and non-vaccine-associated cases of myocarditis and pericarditis. These events will be categorized into two groups for analysis: myocarditis (with or without signs of pericarditis) and pericarditis without myocarditis. Clinical and demographic features, diagnostic testing results, and multi-omics datasets for these cases will be compared to healthy controls and controls with non-vaccine-associated myocarditis.

#### 2.4.2. Thrombosis with Thrombocytopenia Syndrome (TTS)/Vaccine-Induced Immune Thrombocytopenia and Thrombosis (VITT)

The Brighton Collaboration case definition will be applied to both post-vaccine and non-vaccine-associated cases of TTS/VITT [39,40]. This allows differentiation between vaccine-induced TTS/VITT and non-vaccine associated TTS cases. Clinical and demographic features, diagnostic testing results, and multi-omics datasets will be analyzed for PF4+ VITT cases and TTS not meeting VITT criteria, versus healthy vaccinated controls and controls with non-vaccine-associated TTS/VITT-like conditions [39].

#### 2.4.3. Case-control matching

Cases will be matched to healthy controls, recruited through observational vaccine studies and other approaches, who received the same vaccine platform and, where feasible, the same product (e.g., BNT162b2 or mRNA-1273) and did not develop an AEFI. Samples from these controls will be matched to cases at a ratio of at least 1:1 and ideally 3:1 for multi-omics analysis by age group, biological sex, vaccine type, and ancestry.

Cases may also be frequency matched to controls with a non-vaccine-associated TTS/VITT-like condition (e.g., heparin-induced thrombocytopenia [HIT]) or non-vaccine-associated myocarditis or pericarditis, who meet Brighton criteria and have samples collected and stored at the time of their clinical presentation. Matching will be done by age (±5 years), biological sex, ancestry (where possible), and vaccine received (for healthy controls).

#### 2.4.4. Sample collection and processing

Both cases and controls will undergo data and sample collection for multi-omics analyses, including transcriptomics, proteomics, and metabolomics using a standardized protocol. Samples will be captured as close to the onset of an AE as possible, up to 12 weeks after the event, with follow-up samples collected as needed. Participants will also be invited to consent to saliva or blood sampling for DNA extraction and genotyping under the GVDN genomics protocol.

**Fig. 2.**
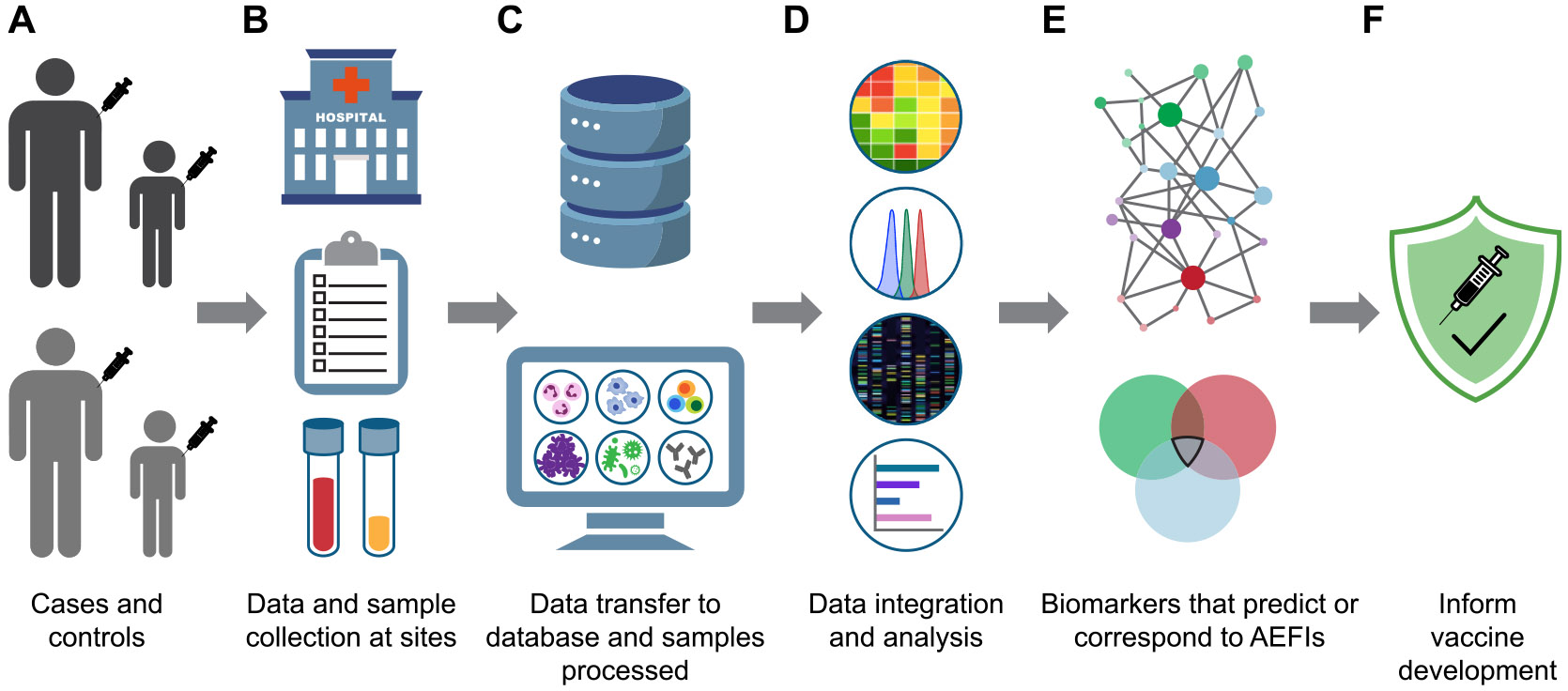
The International Network of Special Immunization Services (INSIS) approach to defining biomarkers of vaccine-associated adverse events. A) AEFI cases and controls are recruited. B) Cases and controls undergo standard assessment, data and sample are collected at INSIS clinical assessment centers. C) Data are transferred to the INSIS central database, and samples are processed at INSIS laboratories for multi-OMICs. D) Integration and analysis of clinical and biological data. E) Biomarkers that predict or correspond to AEFIs will be identified. F) Results will inform vaccine development and personalized vaccination strategies. Abbreviation: AEFI, adverse event following immunization.

Sample collection for cases and controls will be conducted using retrospective residual clinical samples or prospectively at selected sites. At each timepoint (see Table 1), peripheral blood (5–15 ml) will be collected according to protocol. Where processing facilities are available, whole blood (20–30 ml) will be collected for PBMC isolation. Additional sample types, including serum, may be collected according to site-specific or study-specific protocols (refer to INSIS Study SOP in supplementary file 1). Samples for multi-omics analysis will be stored at the participating site until they are ready to be shipped on dry ice or liquid nitrogen to an INSIS laboratory with biobanking facilities, such as the Precision Vaccines Program (PVP), where they will be stored at −80 °C until analysis (Fig. 2). As of May 15, 2025, 7 sites have entered data for 154 cases and 110 controls into the INSIS REDCap database, with >1100 samples (including serum, plasma, PBMC, and saliva) recorded in the INSIS REDCap database. Additionally, > 1600 sample aliquots have been entered into the Laboratory Data Management System (LDMS) [41] sample management system. These numbers will continue to increase as sites enter their data into the INSIS REDCap and LDMS databases.

#### 2.4.5. Core laboratory assays and technologies

**Fig. 3.**
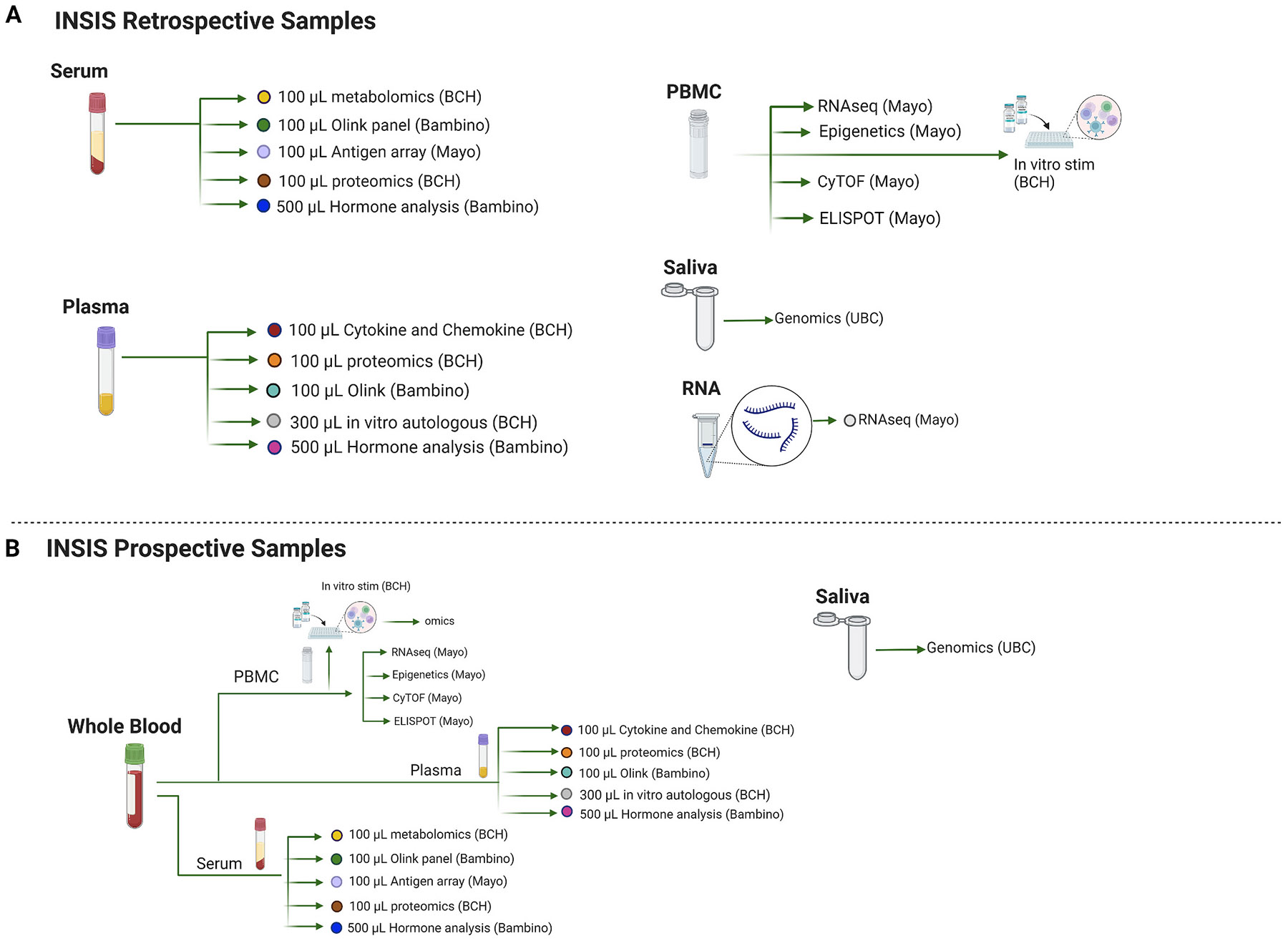
INSIS Sample Processing Pipeline and Core Lab assays. A) The following assay prioritization will be used for retrospective samples: Serum samples will be used to measure metabolomics, proximity extension assay proteomics (Olink), antigen array, proteomics, and hormone analysis. Plasma will be used to measure cytokines and chemokines, proteomics, Olink, human in vitro modeling and hormone analysis. PBMCs will be used for RNAseq or transcriptomics analysis, epigenetics, human in vitro modeling, immunophenotyping using mass cytometry time of flight (CyTOF) and ELISPOT. Saliva will be used for genomics and RNA for transcriptomics analysis. B) For prospective samples; whole blood collected in tubes with an anticoagulant (EDTA preferably) will be processed for PBMCs and plasma samples. Serum will be collected in tubes without any anticoagulant (e.g. SST Greiner tubes). Saliva will be used for genomics. The retrospective samples processing pipeline will be followed for all prospective sample types. Core Labs: Boston Children’s Hospital (BCH), Clinical and Research Unit of Clinical Immunology and Vaccinology, Bambino Gesù Children’s Hospital (Bambino), Mayo Clinic College of Medicine (Mayo), University of British Columbia (UBC).

The following laboratory analyses will be performed on samples collected for this study using a downstream sample-sparing technique for systems biology analyses. Fig. 3 outlines the assay prioritization workflow for the INSIS project (Fig. 3).

### 2.5. Proteomics

#### 2.5.1. Untargeted mass spectrometric profiling

Proteomics will be performed (Steen Lab, Boston Children’s Hospital; Boston, MA) employing a two-pronged approach [42]. First, the plasma proteome will be quantitatively mapped without any depletion considering the important immunomodulatory roles of a sizable fraction of the standard depletion targets such as immunoglobulins and complement pathway components [43,44]. Mapping the vaccine and AEFI-associated changes in abundance for these proteins is important for understanding the pathophysiological processes of these AEFIs. To map the ‘classical’ plasma proteome, the neat plasma will be processed in a high throughput fashion. To map the ‘tissue leakage’ plasma proteome, we will deplete the most abundant plasma samples using perchloric acid, which can be conducted in a high-throughput and cost-efficient manner on thousands of samples [45-47].

The two resulting plasma protein digests will be analyzed by LC-MS in discovery mode using a high-throughput sample delivery and high-performance liquid chromatography (HPLC) system (Evosep One) front-end and a Bruker ion mobility/quadrupole/TOF mass spectrometer (timsTOF HT) back-end to ensure robustness. The instrument will be operated in Data Independent Acquisition (DIA) mode to ensure maximum completeness of the data. All data will be qualitatively and quantitatively analyzed using the Bruker ProteoScape hard- and software environment.

#### 2.5.2. Proximity extension assay (PEA) proteomics (Olink)

Targeted proteomics using Olink’s Proximity Extension Assay (PEA) will be applied to plasma or serum employing a core lab certified workflow. This approach allows for the simultaneous investigation of 92 proteins in 88 samples offering the flexibility to choose from distinct panels (kit target 96, T96) or 45 proteins in 40 samples (kit target 48, T48), using just 1 μl of sample. This technology has also been validated in other biomatrices such as supernatants, serum, tears, biopsies and depending on the kit used, it provides relative (NPX values) or absolute quantitative (pg/ml) results, respectively, in T96 and T48. The kits can also be customized to focus on a specific pathogen/immunological question. This approach will provide additional insights into the protein signatures characterizing AEFIs, comparing them with other conditions, including healthy controls, as previously described in smaller cohorts [48].

#### 2.5.3. Untargeted metabolomics profiling

Metabolomics offers a powerful tool for understanding vaccine safety events by providing comprehensive insights into the biochemical changes associated with vaccination. By detecting specific and consistent metabolic signatures, metabolomics allows for the precise identification of disease states or vaccine responses [49]. For example, alterations in prostaglandin metabolites and eicosanoids, related to hyperinflammation, may help pinpoint the biochemical basis of adverse reactions [50]. Additionally, metabolic dysregulation has been observed in COVID-19 hospitalized patients with severe disease trajectories, including decreased phospholipid components and elevated plasma branched-chain amino acid (BCAA) and urea components captured by untargeted metabolomics [51]. Furthermore, integrating metabolomics with other systems biology platforms can provide a holistic view of the host immune response [49].

Plasma metabolomics assay for the INSIS project will be conducted (Metabolon, Durham, NC) using a previously described workflow [49]. Samples will be randomized into batches, extracted, and prepared for analysis using solvent extraction method [52]. Recovery standards will be added at the initial extraction step to ensure quality control. Proteins will be precipitated with methanol under vigorous shaking and then centrifuged. The resulting supernatants will be divided into five fractions for various analyses, including two reverse phase (RP)/UPLC-MS/MS methods with positive ion mode electrospray ionization (ESI), one RP/UPLC-MS/MS with negative ion mode ESI, one HILIC/UPLC-MS/MS with negative ion mode ESI, and one reserved for backup analysis using high-resolution mass spectrometry. Metabolites will be identified by comparing results to a library of standard metabolites [52] using criteria such as retention index, accurate mass match, and MS/MS scores. Compounds will be categorized according to standards set by the Metabolomics Standards Initiative [53-55]. Appropriate analytical techniques will be used to validate and report metabolites of interest, ensuring accurate and reliable data for further analysis.

### 2.6. Hormone analysis

Considering the higher incidence of myocarditis in males post SARS-CoV-2 infection and vaccination, it has been suggested that androgens may play a potential role in post-COVID-19 vaccination cardiac events [56]. Furthermore, extensive research has been conducted on the impact of androgens on the immune system [57].

Steroid hormone measurement will be conducted on suitable plasma and/or serum samples using liquid chromatography coupled to tandem mass spectrometry (LC-MS/MS). Such analysis will be conducted (Clinical Biochemistry Laboratory, Department of Diagnostic Medicine of the I.R.C.C.S. Bambino Gesù Children’s Hospital) using the Xevo TQ-Smicro Mass Spectrometer of Waters. Sample preparation will be carried out using the CE-IVD certified diagnostic kit of Chromsystems (Munich, Germany) (or equivalent), and samples will be processed on an appropriate platform such as a mass spectrometer equipped with an ACQUITY UPLC I-Class liquid chromatograph that allows the realization of an ultra-high performance and low dispersion liquid chromatography optimized to derive maximum benefits in terms of resolution and sensitivity, and a triple quadrupole that complies with Directive 98/79/EC in all its parts. This type of instrumentation couples ultra-high-performance, low-dispersion chromatography with triple quadrupole used in Multiple Reaction Monitoring (MRM) mode, a specific method developed to detect specific peptides in complex biological mixtures such as human plasma and serum.

### 2.7. SARS-CoV-2 antigen array

Vaccine-associated AEs may have an immunologic trigger. We will use a pathogen proteome array to screen the humoral immune response to SARS-CoV-2 and other relevant coronaviruses in cases and controls to identify differences in humoral immune response generated in those who do and do not experience our selected AEs. Antibody responses to the SARS-CoV-2 proteome will be characterized using a commercial multi-coronavirus protein microarray (Antigen Discovery Inc.; Irvine, CA, USA). The array includes 935 full-length proteins, overlapping protein fragments and overlapping 13–20 aa long peptides from SARS-CoV-2 (WA-1), SARS-CoV, Middle East respiratory syndrome coronavirus (MERSCoV), human coronavirus (HCoV)-NL63, and HCoV-OC43. Proteins will be expressed using an *Escherichia coli* in vitro transcription and translation (IVTT) system (Rapid Translation System, Biotechrabbit, Berlin, Germany). Sera samples will be diluted in PBS, incubated on the microarray. Following removal of the residual serum, bound antibodies a detected using fluorochrome-labelled, anti-human Ig reagents [58-60].

### 2.8. Cytokine and chemokine secretion analysis

Cytokines and chemokines, low-molecular-weight proteins, play crucial roles in the immune response to infection and vaccination. Elevated plasma cytokine levels are associated with severe COVID-19, while vaccination appears to reduce inflammation, potentially mitigating disease severity and mortality. Aberrant cytokine production may contribute to the pathophysiology of severe COVID-19 [61] [62] as well as the vaccine-related AEs [63-65].

The multiplex cytokine and chemokine assay procedures has been previously described [66]and uses the Milliplex Human Cytokine/Chemokine Magnetic Bead Premixed 41 Plex Kit. (cat. #HCYTMAG-60 K-PX41). Cytokines and chemokines measured using the 41-plex Millipore Milliplex Map Kit contains; sCD40L, EGF, FGF-2, Flt-3 ligand, Fractalkine, G-CSF, GM-CSF, GRO (CXCL1), IFN-α2, IFN-γ, IL-1α, IL-1β, IL-1ra, IL-2, IL-3, IL-4, IL-5, IL-6, IL-7, CXCL8, IL-9, IL-10, IL-12 (p40), IL-12 (p70), IL-13, IL-15, IL-17 A, IP-10 (CXCL10), MCP-1 (CCL2), MCP-3 (CCL7), MDC (CCL22), MIP-1α (CCL3), MIP-1β (CCL4), PDGF-AB/BB, RANTES (CCL5), TGF-α, TNF-α, TNF-β, VEGF, Eotaxin (CCL11), and PDGF-AA. Adult plasma samples will be assayed undiluted. The samples will be assayed using a 384-well plate (Corning CellBIND^®^ (cat. #CLS3764)) platform following the manufacturer’s instructions, including the standards and quality controls provided by the kit. Samples will be run and fluorescent signals will be acquired using a Flexmap 3D system with Luminex xPONENT software (Luminex Corp.; Austin, TX, USA). 5-parameter logistic, and exponential functions will be used to fit to the dilution series data per analyte, selecting the best fit function in each case, using Milliplex Analyst Software version 5.1. The curves will be used to determine the lower and upper limits of detection and quantification for each analyte and plate. Analytes that fall below or above these values will be imputed to the lower or upper limit of quantification, respectively. For any given sample and analyte, concentration values will be discarded if readings are <30 beads. Samples with all analytes below the lower limit of detection will be excluded from analysis.

### 2.9. RNA sequencing

AEFIs may be caused by overactive or inappropriate immune responses targeting self-antigens, adverse reactions to viral protein expression, or other dysregulated physiologic responses. These responses must begin with transcriptional changes in the reactive cells and/or tissues. Gene expression analysis of PBMCs will be used to evaluate whether there are transcriptomic signatures associated with AEs following COVID-19 vaccination that can be detected in the peripheral blood. In the case of VITT/TTS, the primary pathology is blood-based and therefore PBMC transcriptomic data will complement the additional analysis of the proteome and metabolome in blood, providing a fuller picture of the molecular mechanisms underlying this adverse event.

PBMC samples will be thawed and cultured under appropriate conditions (e.g., unstimulated or antigen-stimulated) at the Mayo Clinic Vaccine Research Group (MVRG)[ [67]. RNA will be extracted using Qiagen kits and sequencing libraries will be prepared using the TruSeq Stranded mRNA Library Prep kit. mRNA-Seq will be performed in the Mayo Clinic Medical Genome Facility using the Illumina NovaSeq 6000 platform. Flow cell samples will be sequenced as 51 × 2 paired-end reads using HCS v2.0.12 data-collection software or an equivalent platform. Base-calling will be performed using Illumina’s RTA version 1.17.21.3. Gene sequencing data will be aligned using the MAP-RSeq V1 pipeline to the h19 human genome.

### 2.10. Immunophenotyping using mass cytometry time of flight

Immunopathology can be mediated by inappropriate antigen stimulation of lymphocyte populations (e.g., self-reactive T cells) or activation of innate immune cells (e.g., mast cells or basophils triggering anaphylaxis) [68]. Immunophenotyping panels provide an opportunity to comprehensively evaluate both activation status and expansion/contraction of critical immune cell populations that may be responsible for vaccine-related SAEs [69]. CyTOF will be performed with the use of mass cytometry through Mayo Clinic’s Immune Monitoring Core. The Core has a validated panel consisting of 36 antibody markers covering a broad range of leukocyte populations (supplementary file 2) [70].

### 2.11. Genomics analyses

#### 2.11.1. Genome-wide association studies (GWAS)

Extracted DNA from saliva samples will be genotyped with a custom Illumina Global Screening Array (GSA version 3.0 with additional pharmacogenomic content) including genetic variation throughout the genome (500,000 genome-wide markers), further enriched with pharmacogenomic variants including >45,000 variants in core drug absorption, distribution, metabolism, and elimination genes, and > 24,000 variants in major histocompatibility complex (MHC)/HLA gene regions. The array captures both common and rare variants collected from large-scale sequencing projects.

Previously reported candidate genes potentially related to the pathogenesis or biological mechanisms of GBS, TTS/VITT, myocarditis, and pericarditis will be also genotyped by either the custom GSA array or by custom TaqMan genotyping assays. Candidate genes for GBS include, but are not limited to, HLA alleles, IL-10, KIR, TNF-α, CD1, and FcγR. Candidate genes for TTS/VITT include, but are not limited to, F5, F2, PROC, and PROS1. Candidate genes for myocarditis and pericarditis include, but are not limited to, BAG3, DSP, PKP2, RYR2, SCN5A, and TNNI [71,72].

Genotyping will be followed by whole genome imputation of common variants using SHAPEIT (v2) and IMPUTE2 (v2.3.2) in combination with the Phase 31,000 Genomes Project reference panel and imputation of classical HLA alleles and HLA-region variants using SNP2HLA (v1.0.2) in combination with Type 1 Diabetes Genetics Consortium (T1DGC) reference panel. This will yield a final genotyped and imputed dataset of ~10 million variants per sample.

#### 2.11.2. Exome sequencing

From each of the three case groups, 50 of each the most severe AE patients who are categorized as Brighton Collaboration Level One cases of COVID-19-induced GBS, TTS/VITT, or myocarditis/pericarditis will also be selected (a total is 150 for three AEs) to perform exome analyses. This will complement genome-wide genotyping, particularly in protein-coding regions, to identify the most possible disease-causing mutations. The public exome sequencing database, gnomAD, as reference controls will be used to investigate novel and rare genetic variants related to these three specific AEs. Following library preparation with an IDT Capture Expanded Exome Kit, exome sequencing to a mean coverage of 100× will be performed using paired end sequencing (2 × 150 bp) on an Illumina Sequencing platform (NovaSeq platforms). The sequence data will be processed according to GATK Best Practices (v4), using BWA-MEM for alignment of reads to the GRCh38 reference genome on the local high-performance computing cluster. Significant variants identified from the GWAS discoveries will be further validated by genotyping (e.g., TaqMan assays) or sequencing.

### 2.12. Epigenetics

Gene expression is controlled, in part, by epigenetic regulatory features such as DNA methylation. The aim of these experiments is to perform an unbiased measurement of DNA methylation patterns across CpG sites across the genome. DNA methylation patterns that do correlate with vaccine SAEs may provide insights into the biological activities that are dysregulated and contribute to those AEs. Following DNA extraction from PBMC samples, genome-wide methylation patterns will be assessed using either an Illumina Methylation BeadChip or RBBS followed by methyl-Seq.

### 2.13. Human in vitro modeling

To assess whether vaccine-induced molecular signatures may predict AEFIs, where facilities exist, we will collect study participant blood to generate cryopreserved PBMCs and matched autologous plasma. These will be batch shipped to the Precision Vaccines Program (Boston Children’s Hospital, Boston, MA) to conduct human in vitro assays such as PBMCs cultured in 10 % autologous plasma and tissue construct assays as previously described [73-75]. Conditions tested will include vehicle control, agonists of pattern recognition receptors (PRRs) involved in COVID-19 vaccine-induced innate immune responses [76], and authorized/approved COVID-19 vaccines at three dilutions: 1:1000, 1:100 and 1:10 vol/vol. Cellular and soluble fractions will be collected and cryopreserved for downstream systems biology as we have described [77]. Resulting cellular and molecular signatures will be integrated with clinical and immunologic data to provide insights into modeling human in vitro vaccine responses in relation to AEFIs, an approach supported by the United States Food and Drug Administration (FDA) Modernization Act 2.0 [78] aimed at identifying actionable biomarkers to inform future vaccine discovery and development. This human in vitro modeling system provides an ability to recapitulate key aspects of tissue-specific immune responses [70,75,79,80] adding important mechanistic insight to complement in vivo analyses.

## 3. Data management and analytical strategy

### 3.1. Study oversight and reporting

Data are submitted to a central REDCap database hosted and maintained by the Data Management and Analysis Core (DMAC) (PVP, Boston Children’s Hospital; Boston, MA, USA). Participant data will be transferred to the INSIS REDCap database from participating INSIS networks and partners using secure transfer protocols. INSIS sites will be responsible for local quality control and retain ownership of their data. The DMAC will conduct additional data quality checks on the database and maintain a centralized data repository and analytic platform.

Standard forms and labels will be used for sample tracking and linkage to clinical databases. Patient samples will be linked to their record in the database by a unique study ID. The sample management system LDMS [41] will be used to track samples from the point of collection to the analytic laboratories by scanning a sample-specific barcode, which carries a unique sample identifier (sample ID). Date of sample collection, sample type, and volume at each timepoint will be recorded on a sample processing form (SPF) and these metadata will be captured electronically by the central INSIS database. The DMAC will oversee sample tracking and shipping.

Within the framework of executed material transfer and data transfer use agreements (MTAs and DTUAs), and appropriate institutional approvals, INSIS sites will partner with the PVP, Mayo Vaccine Research Group MVRG, Ospedale Pediatrico Bambino Gésu (OPBG), and other INSIS affiliated laboratories to coordinate sample shipping for the multi-omics analysis described above. Integration of clinical and biological data will be conducted using a cloud-based bioinformatic analytic infrastructure. INSIS will work with GVDN to coordinate transfer of clinical data and DNA samples collected at INSIS sites to GVDN (Canadian Pharmacogenomics Network for Drug Safety (CPNDS) lab at University of British Columbia) for processing and analysis.

### 3.2. Data harmonization framework

Harmonized procedural and data processing pipelines across assays are coordinated via the INSIS Data Management Working Group (DMWG) with support from the DMAC. As the cohort is recruited, plans will address missing clinical data and samples and appropriately randomize samples for each assay type to eliminate selection bias and evenly distribute confounding variables. Power calculations will be conducted to ensure that the study is adequately powered to detect significant differences or associations in the data, accounting for potential variability and expected effect sizes. Standardized metadata templates and controlled vocabularies will be applied to ensure data interoperability across sites and assay platforms. As part of our quality control and assurance measures, we recognize that self-reported demographic variables such as gender and race may vary in consistency across study sites. We will incorporate both self-reported and where available, biologically inferred variables (e.g. genetic ancestry, sex chromosome-linked gene expression) during analysis.

**Fig. 4.**
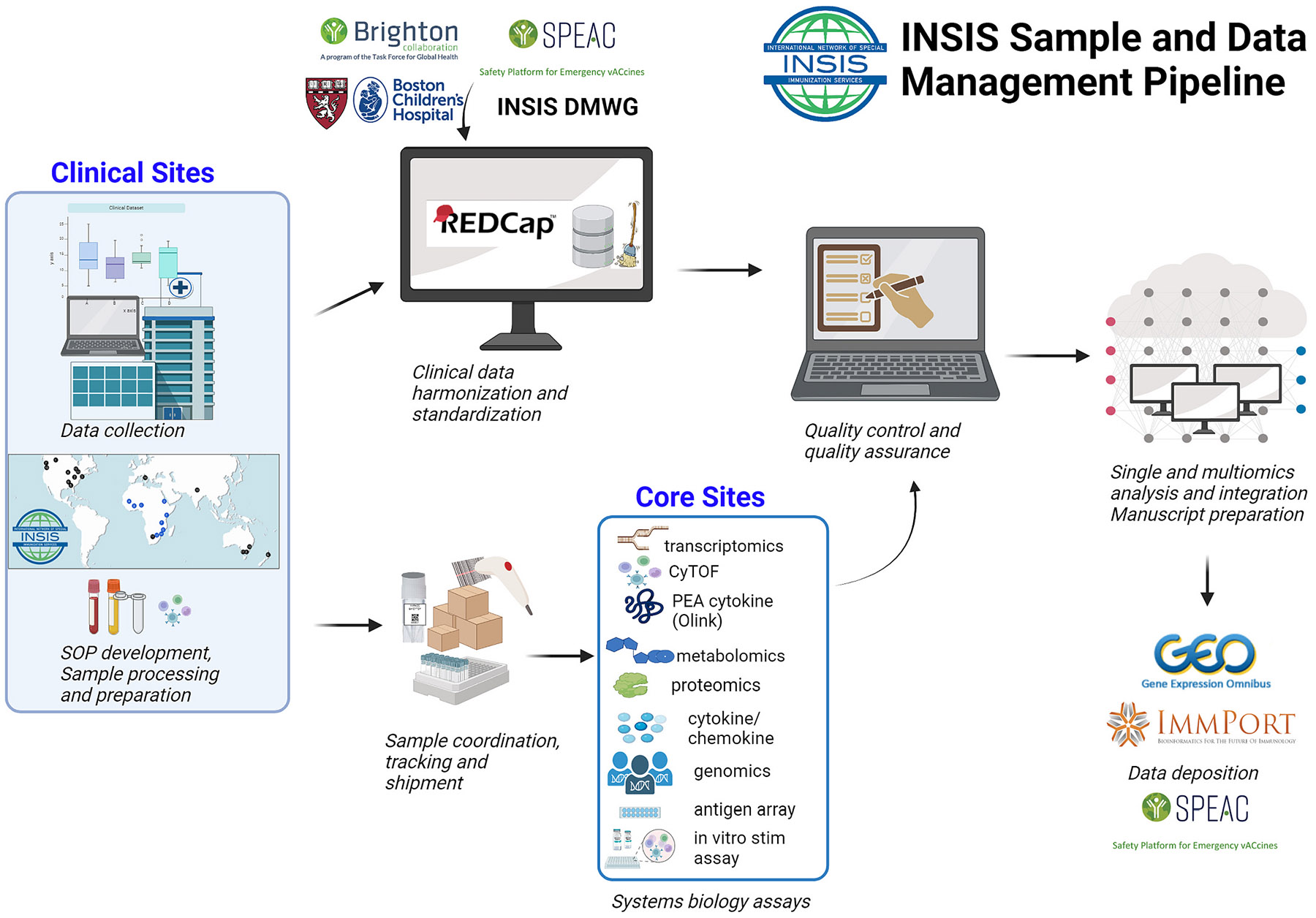
INSIS Sample and Data Management Pipeline. This figure illustrates the comprehensive workflow for sample and data management within the INSIS study. Data collection is performed at various clinical sites, involving the development of standard operating procedures (SOPs), as well as sample processing and preparation. The map illustrates the global locations of these clinical sites. Collected clinical data are harmonized and standardized by the Data Management Working Group (DMWG) using the REDCap platform, in collaboration with the Brighton Collaboration and the Safety Platform for Emergency Vaccines (SPEAC). A rigorous quality control (QC) and quality assurance (QA) process ensures the accuracy and reliability of both data and samples before analysis. Specialized core sites conduct various assays and omics analyses, including transcriptomics, Cytometry by Time-Of-Flight (CyTOF), Proximity Extension Assay (PEA) for cytokine profiling (Olink), metabolomics, proteomics, cytokine/chemokine analysis, genomics, antigen array analysis, and in vitro stimulation assays. The pipeline includes meticulous sample coordination, tracking, and shipment to ensure samples are handled and processed correctly. Single and multi-omics data are analyzed and integrated within a centralized computing environment, facilitating comprehensive data interpretation and manuscript preparation. Final datasets and associated metadata are deposited in public repositories such as NCBI Gene Expression Omnibus (GEO) and ImmPort, ensuring data accessibility for the broader research community.

Once the data are uploaded to the INSIS study database, DMAC data managers and biostatisticians will coordinate and verify quality control (QC) processes for data collected/generated at clinical sites and Core Labs. They will also perform additional quality assurance (QA) to maintain the highest possible accuracy of clinical, immunologic, and systems biology data before reporting and analysis in the centralized cloud computing system (Fig. 4).

### 3.3. Centralized computational platform and data deposition

A cloud computing platform specifically for the INSIS study will be utilized for encrypted, access-controlled data storage and analysis resources [81]. This will include a data and analysis dashboard that allows INSIS investigators to upload, store and analyze both raw and processed computable data within a centralized computing environment. The computing platform will offer a secure environment for developing, testing, and running scripts, as well as performing quality control (QC) and quality assurance (QA) on data generated by the Core Labs. This centralized system will ensure that the INSIS Core Labs adhere to shared data standards and maintain internal consistency, facilitating accurate and integrated data analysis. This setup will be designed to facilitate data sharing and downstream analyses by INSIS investigators and the broader research community, who will be able to access the data and associated metadata via a public data repository such as dbGAP [82] or ImmPort [83] (immport.niaid.nih.gov). Deidentified quality assured published data will be deposited to public repositories according to the funder’s policies.

### 3.4. Analysis of clinical features and outcomes

Clinical features, results of investigations, other exposures or risk factors, and outcomes of the AEFI will be compared within each subgroup by vaccine product, age, biological sex, self-reported gender, and race. Examples of analysis will include multivariable regression analysis which will identify demographic and clinical factors associated with Brighton Collaboration case definition-confirmed TTS/VITT, myocarditis, and pericarditis in cases versus healthy controls. Additionally, we plan to compare factors related to post-vaccination versus non-vaccine associated myocarditis and TTS/VITT versus HIT/VITT-like non-vaccine associated syndromes. Similar approaches will be employed for new AEFIs that emerge as safety signals.

With sufficient sample size, some samples may be used for discovery cohorts and others for replication, with one network (e.g., INSIS) leading an omics discovery analysis and providing samples for the replication of another network’s analyses. Similar sample size and matching approaches will be used for new AEFI targets. INSIS will partner with GVDN to recruit cases and controls for genomics analysis in GVDN-led studies.

### 3.5. Integrated multi-omics analysis

Multi-omics data from patients with well-defined phenotypes (e.g., Brighton level 1 TTS/VITT) and controls will be analyzed using data integration approaches such as MultiOmics Factors Analysis (MOFA) and Data Integration Analysis for Biomarker Discovery using Latent Components (DIABLO) as previously described [84,85]. MOFA is a computational method used to integrate and analyze multi-omics datasets which distinguishes between patterns that are shared across different omics layers and those that are specific to individual layers [84]. DIABLO identifies key drivers associated with the response variable of interest across all input data matrices jointly. Cross-validation will determine the optimal model hyperparameters (number of components, features per component) and estimate the model’s ability to generalize to new data. Selected model features will undergo pathway over-representation analysis against the Reactome pathways database (via MSigDB) and blood transcriptional module (BTM) annotated gene set libraries, with *p*-values adjusted to control false discovery rate (FDR).

Multi-omics data will be compared within groups over time (e.g., myocarditis/TTS/VITT onset vs post-recovery) and between cases and controls to identify differences in basal levels of analytes in cases (using baseline samples: pre-vaccination, post-recovery/ ≥3 months postvaccination), as well as at time of myocarditis/TTS/VITT diagnosis (and similar timepoints post-vaccination in controls). Analyte levels in blood/plasma will also be compared to normal ranges in adults where available. Two-way comparisons will be performed between cases and healthy controls vs cases and controls with non-vaccine associated myocarditis or HIT.

## 4. Governance and organizational structure

INSIS is a global consortium focused on AEFIs and involving key clinical consultation services including the SIC Network, AEFI-CAN, members of the US Clinical Immunization Safety Assessment (CISA) network, Brighton Collaboration, GVDN, African Leadership in Vaccinology Expertise (ALIVE) network, experts in systems immunology (PVP, Boston Children’s Hospital; MVRG, OPBG, Rome, IT) and pharmacogenomics (University of British Columbia and BC Children’s Hospital Research Institute), and experts in pharmacogenomics and global vaccine policy (Global Healthcare Consulting and University of Washington). INSIS is managed by the Network Management Office (NMO) at the University of Alberta, under the leadership of the Nominated Principal Investigator (NPI) and Brighton Co-lead. The NMO coordinates operations, including finance, contracts, and communications, and interacts with the Task Force for Global Health, which hosts the Brighton Collaboration.

The Steering Committee, comprising representatives from key clinical networks and experts in vaccine safety and systems biology, oversees network governance, project progress, and funding. Monthly meetings facilitated by the NMO ensure alignment with project milestones. Specialized Working Groups, such as Data Management and OMICs, also meet monthly to support project deliverables. INSIS holds monthly calls open to all members to discuss and plan project progress and feature guest speakers on vaccine safety topics.

### 4.1. Scientific advisory board and dissemination strategy

INSIS will establish a Scientific Advisory Board (SAB) with diverse representation from funders, regulators, public health experts, and stakeholders, particularly from LMICs. The SAB will provide guidance on research priorities and ensure results are aligned with stakeholder needs and translated into policy.

INSIS will disseminate findings through its website, reports, presentations, open-access publications, and conferences. A Publication and Presentation Policy, following ICMJE guidelines, governs authorship and dissemination. Results will also be shared with participants and the public through institutional websites, media channels, and social media, with support from partner organizations’ communications teams.

## 5. Conclusions

The establishment of INSIS marks a significant advancement in the global effort to understand and mitigate rare AEFIs associated with COVID-19 vaccination. By integrating clinical data with advanced multi-omics technologies, INSIS provides a comprehensive platform for investigating the underlying mechanisms of AEFIs such as COVID vaccine-induced immune thrombocytopenia and thrombosis (VITT) and myocarditis. The rigorous data management and quality assurance processes employed by INSIS will ensure the accuracy and reliability of the collected data, facilitating robust analyses and meaningful conclusions. This approach will not only enhance our understanding of these conditions but also will inform vaccine development and development of personalized vaccination strategies, ultimately aiming to improve public health outcomes.

As the work progresses and the network continues to expand, INSIS aims to apply the methodology described herein to new AEFIs and additional vaccines targeting diverse threats. INSIS’ collaborative framework and cutting-edge methodologies will remain crucial in addressing emerging vaccine safety signals. INSIS’s efforts highlight the importance of global collaboration in vaccine safety research and emphasize the potential of integrating clinical, immunologic, and multi-omics analysis to drive scientific discoveries and inform public health policies.

## Supplementary Material

Suppl file 2

Suppl file 1

## Figures and Tables

**Table 1:** Schedule of study procedures, prospectively enrolled cases and controls for multi-OMICs analysis. *†, ‡

	During adverse event/onset myocarditis/VITT/TTS	2–8 weeks after event onset†	~12–20 weeks after onset	~6–24 months after onset
Cases and controls with non-vaccineassociated disease	Consent (if possible) Data collection Blood collection*	Consent‡ Data collection Blood collection	Consent (if not yet obtained) ‡ Data collection Blood collection	Data collection Blood collection
	Pre-vaccination (if available)	~3–90 days post vaccination**	~12–20 weeks post vaccination	~6–24 months post vaccination
Controls	Consent (if possible) Data collection Blood collection**	Consent‡ Data collection Blood collection	Consent (if not yet obtained) ‡ Data collection Blood collection	Data collection Blood collection

* Participants may be included if multi-OMICs samples are available from only 1 timepoint but 2 or more timepoints are preferred within these approximate timeframes (preferably an acute sample and follow up sample). In some cases an early follow up sample and late follow up sample (e.g., 3 and > 6 months) will be acceptable. A maximum of 90 ml of blood will be drawn on children <16 years of age.

† At time of first specialist assessment (e.g., cardiology, special immunization clinic).

‡ If not consented at presentation, consent will be obtained at time of specialist assessment for retrieval of residual serum/plasma from initial presentation.

** Aim for 3–7 days post-vaccination for controls matched to myocarditis cases; 5–42 days post-vaccination for controls matched to VITT cases, longer timeframes may be appropriate for additional AEFI of interest.

## Data Availability

No data are included in this paper, as it is a study protocol. This document outlines the planned methodology and study design. Any data that will be generated during the course of the research will be shared in accordance with our study protocol guidelines for data deposition and will be accessible upon reasonable request. The data will be deposited in a public repository once the study is completed and all necessary ethical approvals are obtained.

## INSIS Members who contributed to this publication are:

**Baylor College of Medicine, Molecular Virology and Microbiology, Houston, TX, 77030, USA:** Jennifer Whitaker, Kristen Sexson.

**Boston Children’s Hospital, Boston, MA, 02115, USA:** Al Ozonoff, Asimenia Angelidou, Ana C Chang, Annmarie Hoch, Caitlin Syphurs, Caitlyn McLoughlin, Kerry McEnaney, Hanno Steen, Jing Chen, Kinga K. Smolen, Mahitha Donthireddy, Sarah K Steltz, Simon van Haren, Joann Diray-Arce, Ofer Levy.

**Brighton Collaboration, Task Force for Global Health, Decatur, GA 30030, USA:** Dale Nordenberg, Robert T. Chen.

**Channing Division of Medicine, Brigham and Women’s Hospital, Boston, MA, 02115, USA:** Jessica Lasky-Su.

**Department of Cardiovascular Medicine, Mayo Clinic, Rochester, MN 55905, USA:** Tahir S. Kafil.

**Global Vaccine Data Network, Auckland, 1142, New Zealand:** Helen Petousis-Harris, Steve Black.

**Johns Hopkins Bloomberg School of Public Health, Baltimore, MD, 20912, USA:** Kawsar Talaat.

**KEMRI-Wellcome Trust Research Programme, Kilifi, Kenya, South Africa:** Eunice Wangeci Kagucia, Samuel Sang.

**Massachusetts General Research Institute, Boston, MA, 02114, USA:** Lael Yonker.

**McMaster University, Hamilton, ON, L8S 4 K1, Canada:** Donald Arnold, Ishac Nazy,Meera Karunakaran, Rumi Clare.

**Monash University and Alfred Health, Melbourne, VIC, 3004, Australia:** Huyen Tran.

**Murdoch Children’s Research Institute (MCRI_AEFI-CAN), Melbourne, VIC, 3052, Australia:** Annette Alafaci, Jim Buttery, Nigel Crawford.

**Odense University Hospital, Odense, 5000, Denmark:** Lennart Friis-Hansen.

**Research Laboratories, Bambino Gesù Children’s Research Hospital, IRCCS, Rome, 00165, Italy:** Donato Amodio, Emma Concetta Manno, Paolo Palma, Veronica Santilli.

**Thriive, Bronx, NY:** Ariel Zadok, Dale Nordenberg.

**The Hospital for Sick Children, University of Toronto, Toronto, ON, M5G 1 × 8, Canada:** Amy Xu, Olivia Garisto, Aaron Mulivor, Ashish Nambiar, Trang Duong, Rae S. M. Yeung.

**Uniformed Services University of the Health Sciences, Bethesda, MD, 20814, USA:** Renata Engler.

**University Medical Center Utrecht, Utrecht, 3584, Netherlands:** Fariba Ahmadizar, Sima Mohammadi.

**University of Alberta, Edmonton, AB, T6G 1C9, Canada:** Amanda Wilson, Sara Moradipoor, Gavin Oudit, Karina A. Top.

**University of Auckland, 1142, New Zealand:** Helen Petousis-Harris.

**University of British Columbia, Vancouver, BC, V6T 1Z4, Canada:** Wan-Chun Chang, Bruce Carleton.

**University of Ottawa Heart Institute, Ottawa, ON, K1W 4 W7, Canada:** Ermina Moga, Kimberly Kidder, Liyong Zhang, Peter Liu.

**University of Sydney, Sydney, NSW, 2006, Australia:** Nicholas Wood, Vivien Chen.

**University of Washington, Seattle, WA, 98915, USA and Global Healthcare Consulting:** Sonali Kochhar.

**UT Southwestern Medical Center, Dallas, TX, 75390, USA:** Ann Marie Navar.

**Vaccine Research Group, Mayo Clinic, Rochester, MN, 55905, USA:** Richard B. Kennedy, Inna G. Ovsyannikova, Gregory A. Poland.

**Vanderbilt University Medical Center, Nashville, TN, 37232, USA:** Emily Mitchell, Kathryn Edwards, Sandra Yoder, Shelly McGehee, C. Buddy Creech.

**Walter Reed National Military Medical Center, Bethesda, MD, 20889, USA:** Jay Montgomery.

**Weill Cornell Medical College - Pediatrics, New York, NY, 10065, USA:** James B Bussel.

**University of the Witwatersrand, Vaccines and Infectious Diseases Analytics (VIDA) Research Unit, Johannesburg, 2050, South Africa:** Kimberley Gutu, Ziyaad Dangor, Clare L. Cutland.

## Appendix A. Supplementary data

Supplementary data to this article can be found online at https://doi.org/10.1016/j.vaccine.2025.127504.

## Abbreviations:

AEFI: Adverse Event Following Immunization
AESI: Adverse Event of Special Interest
VITT: Vaccine-Induced Immune Thrombocytopenia and Thrombosis
TTS: Thrombosis with Thrombocytopenia Syndrome
INSIS: The International Network of Special Immunization Services
PF4: Anti-platelet Factor 4
ITP: Immune Thrombocytopenic Purpura
GBS: Guillain-Barré Syndrome
SAE: Serious Adverse Event
HIT: Heparin Induced Thrombocytopenia
MACE: Major Adverse Cardiac Events
LVEF: Left Ventricular Dysfunction
RSV: Respiratory Syncytial Virus
PBMC: Peripheral Blood Mononuclear Cells
MERSCoV: Middle East Respiratory Syndrome Coronavirus
HCoV: Human Coronavirus
PRR: Pattern Recognition Receptor
SIC: Special Immunization Clinic Network
AEFI-CAN: Australian Adverse Event Following Immunization-Clinical Assessment Network
CISA: US Clinical Immunization Safety Assessment Network
GVDN: Global Vaccine Data Network
ALIVE: African Leadership in Vaccinology Expertise Network
PVP: Precision Vaccines Program
MVRG: Mayo Clinic Vaccine Research Group
OPBG: Bambino Gesù Children’s Hospital
UBC: University of British Columbia
SickKids: The Hospital for Sick Children
SAB: Scientific Advisory Board
ICMJE: International Committee of Medical Journal Editors
CDC: Centers for Disease Control and Prevention
WHO: World Health Organization
LMIC: Low and Middle Income Country
NIH: United States National Institutes of Health
DMAC: Data Management and Analysis Core
NPI: Nominated Principal Investigator
NMO: Network Management Office
SPEAC: Safety Platform for Emergency Vaccines
LC-MS: Liquid Chromatography coupled to tandem Mass Spectrometry
HPLC: High-Performance Liquid Chromatography
DIA: Data Independent Acquisition
PEA: Proximity Extension Assay
BCAA: Branched-Chain Amino Acid
RP: Reverse Phase
UPLC-MS: Ultra-Performance Liquid Chromatography-Mass Spectrometry
ESI: Electrospray Ionization
HILIC: Hydrophilic Interaction Liquid Chromatography
MRM: Multiple Reaction Monitoring
IVTT: In Vitro Transcription and Translation
CyTOF: Mass Cytometry Time of Flight
GWAS: Genome-Wide Association Studies
GSA: Global Screening Array
MHC: Major Histocompatibility Complex
GATK: Genome Analysis Toolkit
MOFA: MultiOmics Factors Analysis
DIABLO: Data Integration Analysis for Biomarker Discovery using Latent Components
BTM: Blood Transcriptional Module
FDR: False Discovery Rate
GEO: Gene Expression Omnibus
SOP: Standard Operation Procedure
CRF: Case Report Form
SPF: Sample Processing Form
MTAs: Material Transfer Agreements
DTUAs: Data Transfer Use Agreements
QC: Quality Control
QA: Quality Assurance

## References

[1] Wilson GS. The hazards of immunization. Athlone P; 1967.

[2] Chen RT, Shimabukuro TT, Martin DB, Zuber PLF, Weibel DM, Sturkenboom M. Enhancing vaccine safety capacity globally: a lifecycle perspective. Vaccine 2015;33:D46–54. 26433922 10.1016/j.vaccine.2015.06.073 PMC4663114

[3] Salmon DA, Chen RT, Black S, Sharfstein J. Lessons learned from COVID-19, H1N1, and routine vaccine pharmacovigilance in the United States: a path to a more robust vaccine safety program. Expert Opin Drug Saf 2024;23(2):161–75. 38343204 10.1080/14740338.2024.2305707

[4] Canada H. Drug and vaccine authorizations for COVID-19: List of authorized drugs, vaccines and expanded indications. Available from, https://www.canada.ca/en/health-canada/services/drugs-health-products/covid19-industry/drugs-vaccines-treatments/authorization/list-drugs.html; 2024.

[5] UK Health Security Agency. COVID-19: the green book. Chapter 14a: SARS-CoV-2 (COVID-19). 2020. p. 1–24.

[6] Watson OJ, Barnsley G, Toor J, Hogan AB, Winskill P, Ghani AC. Global impact of the first year of COVID-19 vaccination: a mathematical modelling study. Lancet Infect Dis 2022;22(9):1293–302. 35753318 10.1016/S1473-3099(22)00320-6 PMC9225255

[7] Lau CL, Mayfield HJ, Sinclair JE, Brown SJ, Waller M, Enjeti AK, Risk-benefit analysis of the AstraZeneca COVID-19 vaccine in Australia using a Bayesian network modelling framework. Vaccine 2021;39(51). 10.1016/j.vaccine.2021.10.079 PMC8566665 34810000

[8] Chandler RE, Balakrishnan MR, Brasseur D, Bryan P, Espie E, Hartmann K, Collaboration within the global vaccine safety surveillance ecosystem during the COVID-19 pandemic: lessons learnt and key recommendations from the COVAX vaccine safety working group. BMJ Glob Health 2024;9(3):e014544. 10.1136/bmjgh-2023-014544 PMC10921508 38453518

[9] Hippisley-Cox J, Patone M, Mei XW, Saatci D, Dixon S, Khunti K, Risk of thrombocytopenia and thromboembolism after covid-19 vaccination and SARS-CoV-2 positive testing: self-controlled case series study. BMJ 2021;n1931. 10.1136/bmj.n1931 PMC8388189 34446426

[10] Patone M, Handunnetthi L, Saatci D, Pan J, Katikireddi SV, Razvi S, Neurological complications after first dose of COVID-19 vaccines and SARS-CoV-2 infection. Nat Med 2021;27(12):2144–53. 34697502 10.1038/s41591-021-01556-7 PMC8629105

[11] Schultz NH, Sorvoll IH, Michelsen AE, Munthe LA, Lund-Johansen F, Ahlen MT, Thrombosis and thrombocytopenia after ChAdOx1 nCoV-19 vaccination. N Engl J Med 2021;384(22):2124–30. 33835768 10.1056/NEJMoa2104882 PMC8112568

[12] Greinacher A, Thiele T, Warkentin TE, Weisser K, Kyrle PA, Eichinger S. Thrombotic thrombocytopenia after ChAdOx1 nCov-19 vaccination. N Engl J Med 2021;384(22):2092–101. 33835769 10.1056/NEJMoa2104840 PMC8095372

[13] Agency EM. Astrazeneca’s COVID-19 vaccine: EMA finds possible link to very rare cases of unusual blood clots with low blood platelets. cited 2024. Available from, https://www.ema.europa.eu/en/news/astrazenecas-covid-19-vaccine-ema-finds-possible-link-very-rare-cases-unusual-blood-clots-low-blood-platelets; 2021.

[14] MHRA issues new advice, concluding a possible link between COVID-19 Vaccine AstraZeneca and extremely rare, unlikely to occur blood clots [press release]. 2021.

[15] Joint CDC and FDA Statement on Johnson & Johnson COVID-19 Vaccine [press release]. 2021.

[16] Huynh A, Kelton JG, Arnold DM, Daka M, Nazy I. Antibody epitopes in vaccine-induced immune thrombotic thrombocytopaenia. Nature 2021;596(7873):565–9. 34233346 10.1038/s41586-021-03744-4

[17] Greinacher A, Langer F, Makris M, Pai M, Pavord S, Tran H, Vaccine-induced immune thrombotic thrombocytopenia (VITT): update on diagnosis and management considering different resources. J Thromb Haemost 2022;20(1):149–56. 34693641 10.1111/jth.15572 PMC8646430

[18] Shay DK, Shimabukuro TT, DeStefano F. Myocarditis occurring after immunization with mRNA-based COVID-19 vaccines. JAMA Cardiol 2021;6(10):1115–7. 34185047 10.1001/jamacardio.2021.2821

[19] Montgomery J, Ryan M, Engler R, Hoffman D, McClenathan B, Collins L, Myocarditis following immunization with mRNA COVID-19 vaccines in members of the US military. JAMA Cardiol 2021;6(10):1202–6. 34185045 10.1001/jamacardio.2021.2833 PMC8243257

[20] Rosner CM, Genovese L, Tehrani BN, Atkins M, Bakhshi H, Chaudhri S, Myocarditis temporally associated with COVID-19 vaccination. Circulation 2021;144(6):502–5. 34133885 10.1161/CIRCULATIONAHA.121.055891 PMC8340723

[21] Larson KF, Ammirati E, Adler ED, Cooper LT Jr, Hong KN, Saponara G, Myocarditis after BNT162b2 and mRNA-1273 vaccination. Circulation 2021;144(6):506–8. 34133884 10.1161/CIRCULATIONAHA.121.055913 PMC8340725

[22] Marshall M, Ferguson ID, Lewis P, Jaggi P, Gagliardo C, Collins JS, Symptomatic acute myocarditis in 7 adolescents after Pfizer-BioNTech COVID-19 vaccination. Pediatrics 2021;148(3). 10.1542/peds.2021-052478 34088762

[23] Patone M, Mei XW, Handunnetthi L, Dixon S, Zaccardi F, Shankar-Hari M, Risk of myocarditis after sequential doses of COVID-19 vaccine and SARS-CoV-2 infection by age and sex. Circulation 2022;146(10):743–54. 35993236 10.1161/CIRCULATIONAHA.122.059970 PMC9439633

[24] Klein NP, Lewis N, Goddard K, Fireman B, Zerbo O, Hanson KE, Surveillance for adverse events after COVID-19 mRNA vaccination. JAMA 2021;326(14):1390–9. 34477808 10.1001/jama.2021.15072 PMC8511971

[25] Oster ME, Shay DK, Su JR, Gee J, Creech CB, Broder KR, Myocarditis cases reported after mRNA-based COVID-19 vaccination in the US from December 2020 to august 2021. JAMA 2022;327(4):331. 35076665 10.1001/jama.2021.24110 PMC8790664

[26] Kracalik I, Oster ME, Broder KR, Cortese MM, Glover M, Shields K, Outcomes at least 90 days since onset of myocarditis after mRNA COVID-19 vaccination in adolescents and young adults in the USA: a follow-up surveillance study. The Lancet Child & Adolescent Health 2022;6(11):788–98. 36152650 10.1016/S2352-4642(22)00244-9 PMC9555956

[27] Ronk AJ, Lloyd NM, Zhang M, Atyeo C, Perrett HR, Mire CE, A Lassa virus mRNA vaccine confers protection but does not require neutralizing antibody in a guinea pig model of infection. Nat Commun 2023;14(1). 10.1038/s41467-023-41376-6 PMC10497546 37699929

[28] Dunkle LM, Kotloff KL, Gay CL, Anez G, Adelglass JM, Barrat Hernandez AQ, Efficacy and safety of NVX-CoV2373 in adults in the United States and Mexico. N Engl J Med 2022;386(6):531–43. 34910859 10.1056/NEJMoa2116185 PMC8693692

[29] FDA. Novavax COVID-19 Vaccine, Adjuvanted. Available from, https://www.fda.gov/vaccines-blood-biologics/coronavirus-covid-19-cber-regulated-biologics/novavax-covid-19-vaccine-adjuvanted; 2023.

[30] Top KA, Chen RT, Levy O, Ozonoff A, Carleton B, Crawford NW, Advancing the science of vaccine safety during the coronavirus disease 2019 (COVID-19) pandemic and beyond: launching an international network of special immunization services. Clin Infect Dis 2022;75(Supplement_1). S11–S7. 35680552 10.1093/cid/ciac407 PMC9376276

[31] Luk A, Clarke B, Dahdah N, Ducharme A, Krahn A, McCrindle B, Myocarditis and pericarditis after COVID-19 mRNA vaccination: practical considerations for care providers. Can J Cardiol 2021;37(10):1629–34. 34375696 10.1016/j.cjca.2021.08.001 PMC8349442

[32] Global Vaccine Data Network. Genomics of COVID-19 vaccine-related adverse events [cited 2024].. Available from: https://www.globalvaccinedatanetwork.org/ourwork/genomics-covid-19-vaccine-related-adverse-events; 2024.

[33] Poland GA, Kennedy RB, McKinney BA, Ovsyannikova IG, Lambert ND, Jacobson RM, Vaccinomics, adversomics, and the immune response network theory: individualized vaccinology in the 21st century. Semin Immunol 2013;25(2):89–103. 23755893 10.1016/j.smim.2013.04.007 PMC3752773

[34] Soni D, Van Haren SD, Idoko OT, Evans JT, Diray-Arce J, Dowling DJ, Towards precision vaccines: lessons from the second international precision vaccines conference. Front Immunol 2020;11:590373. 33178222 10.3389/fimmu.2020.590373 PMC7593811

[35] Zhou X, Zhang X, Larson HJ, de Figueiredo A, Jit M, Fodeh S, Spatiotemporal trends in COVID-19 vaccine sentiments on a social media platform and correlations with reported vaccine coverage. Bull World Health Organ 2024;102(1):32–45. 38164328 10.2471/BLT.23.289682 PMC10753281

[36] Kochhar S, Izurieta HS, Chandler RE, Hacker A, Chen RT, Levitan B. Benefit-risk assessment of vaccines. Vaccine 2024;42(4):969–71. 37563049 10.1016/j.vaccine.2023.07.041

[37] Sexson Tejtel SK, Munoz FM, Al-Ammouri I, Savorgnan F, Guggilla RK, Khuri-Bulos N, Myocarditis and pericarditis: case definition and guidelines for data collection, analysis, and presentation of immunization safety data. Vaccine 2022;40(10):1499–511. 35105494 10.1016/j.vaccine.2021.11.074

[38] Brighton Collaboration. Case Definitions. Available from: https://brightoncollaboration.org/case-definitions/; Accessed 2024.

[39] Schönborn L, Pavord S, Chen VMY, Pai M, Gwarzo DH, Buttery J, Thrombosis with thrombocytopenia syndrome (TTS) and vaccine-induced immune thrombocytopenia and thrombosis (VITT): Brighton collaboration case definitions and guidelines for data collection, analysis, and presentation of immunisation safety data. Vaccine 2024;42(7):1799–811. 38302339 10.1016/j.vaccine.2024.01.045

[40] Collaboration B. Thrombosis with thrombocytopenia syndrome (TTS) and vaccine-induced immune thrombocytopenia and thrombosis (VITT). cited 2024. Available from, https://brightoncollaboration.org/thrombosis-with-thrombocytopenia-syndrome-vaccine-induced-immune-thrombocytopenia-and-thrombosis/; 2024. 10.1016/j.vaccine.2024.01.045 38302339

[41] Frontier Science Foundation. LDMS: Laboratory Data Management System. Available from: https://www.ldms.org/. Accessed 2025.

[42] Bennike TB, Steen H. High-throughput parallel proteomic sample preparation using 96-well Polyvinylidene fluoride (PVDF) membranes and C18 purification plates. Methods Mol Biol 2017;1619:395–402. 28674899 10.1007/978-1-4939-7057-5_27

[43] Anderson NL, Anderson NG. The human plasma proteome: history, character, and diagnostic prospects. Mol Cell Proteomics 2002;1(11):845–67. 12488461 10.1074/mcp.r200007-mcp200

[44] Fejgin M, Jaffe R, Cohen I, Ben-Aderet N. Control of prostaglandin induced uterine hyperactivity with intravenous ritodrine. Int J Gynaecol Obstet 1989;30(2):177–8. 2572490 10.1016/0020-7292(89)90314-7

[45] Li J, Wang L, Chen Z, Xie R, Li Y, Hang T, Development and validation of a rapid HPLC method for the determination of cefdinir in beagle dog plasma integrated with an automatic on-line solid-phase extraction following protein precipitation in the 96-well plate format. J Chromatogr B Anal Technol Biomed Life Sci 2012;895–896:83–8. 10.1016/j.jchromb.2012.03.018 22503733

[46] Zougman A, Wisniewski JR. Beyond linker histones and high mobility group proteins: global profiling of perchloric acid soluble proteins. J Proteome Res 2006;5(4):925–34. 16602700 10.1021/pr050415p

[47] Viode A, van Zalm P, Smolen KK, Fatou B, Stevenson D, Jha M, A simple, time- and cost-effective, high-throughput depletion strategy for deep plasma proteomics. Sci Adv 2023;9(13):eadf9717. 36989362 10.1126/sciadv.adf9717 PMC10058233

[48] Amodio D, Pascucci GR, Cotugno N, Rossetti C, Manno EC, Pighi C, Similarities and differences between myocarditis following COVID-19 mRNA vaccine and multiple inflammatory syndrome with cardiac involvement in children. Clin Immunol 2023;255:109751. 37660743 10.1016/j.clim.2023.109751

[49] Diray-Arce J, Conti MG, Petrova B, Kanarek N, Angelidou A, Levy O. Integrative metabolomics to identify molecular signatures of responses to vaccines and infections. Metabolites 2020;10(12). 10.3390/metabo10120492 PMC7760881 33266347

[50] Zhuang CL, Lin ZJ, Bi ZF, Qiu LX, Hu FF, Liu XH, Inflammation-related adverse reactions following vaccination potentially indicate a stronger immune response. Emerg Microbes Infect 2021;10(1):365–75. 33583360 10.1080/22221751.2021.1891002 PMC7928063

[51] Diray-Arce J, Fourati S, Doni Jayavelu N, Patel R, Maguire C, Chang AC, Multi-omic longitudinal study reveals immune correlates of clinical course among hospitalized COVID-19 patients. Cell Rep Med 2023;4(6):101079. 37327781 10.1016/j.xcrm.2023.101079 PMC10203880

[52] Evans AM, DeHaven CD, Barrett T, Mitchell M, Milgram E. Integrated, nontargeted ultrahigh performance liquid chromatography/electrospray ionization tandem mass spectrometry platform for the identification and relative quantification of the small-molecule complement of biological systems. Anal Chem 2009;81(16):6656–67. 19624122 10.1021/ac901536h

[53] Spicer RA, Salek R, Steinbeck C. A decade after the metabolomics standards initiative it’s time for a revision. Sci Data 2017;4:170138. 29989594 10.1038/sdata.2017.138 PMC6038898

[54] Sumner LW, Amberg A, Barrett D, Beale MH, Beger R, Daykin CA, Proposed minimum reporting standards for chemical analysis chemical analysis working group (CAWG) metabolomics standards initiative (MSI). Metabolomics 2007;3(3):211–21. 24039616 10.1007/s11306-007-0082-2 PMC3772505

[55] Sansone SA, Fan T, Goodacre R, Griffin JL, Hardy NW, Kaddurah-Daouk R, The metabolomics standards initiative. Nat Biotechnol 2007;25(8):846–8. 10.1038/nbt0807-846b 17687353

[56] Le Vu S, Bertrand M, Jabagi MJ, Botton J, Drouin J, Baricault B, Age and sex-specific risks of myocarditis and pericarditis following Covid-19 messenger RNA vaccines. Nat Commun 2022;13(1):3633. 35752614 10.1038/s41467-022-31401-5 PMC9233673

[57] Prall SP, Muehlenbein MP. DHEA modulates immune function: a review of evidence. Vitam Horm 2018;108:125–44. 30029724 10.1016/bs.vh.2018.01.023

[58] Bode L, Bertrand K, Najera JA, Furst A, Honerkamp-Smith G, Shandling AD, Characterization of SARS-CoV-2 antibodies in human milk from 21 women with confirmed COVID-19 infection. Pediatr Res 2023;93(6):1626–33. 36434204 10.1038/s41390-022-02360-w PMC9702863

[59] Camerini D, Randall AZ, Trappl-Kimmons K, Oberai A, Hung C, Edgar J, Mapping SARS-CoV-2 antibody epitopes in COVID-19 patients with a multi-coronavirus protein microarray. Microbiol Spectr 2021;9(2):e0141621. 10.21203/rs.3.rs-1083825/v1. 34704808 PMC8549749

[60] Sasson JM, Campo JJ, Carpenter RM, Young MK, Randall AZ, Trappl-Kimmons K, Diverse humoral immune responses in younger and older adult COVID-19 patients. mBio 2021;12(3):e0122921. 34182775 10.1128/mBio.01229-21 PMC8262923

[61] Merad M, Subramanian A, Wang TT. An aberrant inflammatory response in severe COVID-19. Cell Host Microbe. 2021;29(7):1043–7. 10.1016/j.chom.2021.06.018. 34265243 PMC8279571

[62] Hawerkamp HC, Dyer AH, Patil ND, Mcelheron M, O’Dowd N, O’Doherty L, Characterisation of the pro-inflammatory cytokine signature in severe COVID-19. Front Immunol 2023;14. 10.3389/fimmu.2023.1170012 PMC10101230 37063871

[63] Barmada A, Klein J, Ramaswamy A, Brodsky NN, Jaycox JR, Sheikha H, Cytokinopathy with aberrant cytotoxic lymphocytes and profibrotic myeloid response in SARS-CoV-2 mRNA vaccine-associated myocarditis. Sci Immunol 2023;8(83):eadh3455. 37146127 10.1126/sciimmunol.adh3455 PMC10468758

[64] Murata K, Nakao N, Ishiuchi N, Fukui T, Katsuya N, Fukumoto W, Four cases of cytokine storm after COVID-19 vaccination: case report. Front Immunol 2022;13:967226. 36045681 10.3389/fimmu.2022.967226 PMC9420842

[65] Bergamaschi C, Terpos E, Rosati M, Angel M, Bear J, Stellas D, Systemic IL-15, IFN-γ, and IP-10/CXCL10 signature associated with effective immune response to SARS-CoV-2 in BNT162b2 mRNA vaccine recipients. Cell Rep 2021;36(6):109504. 34352226 10.1016/j.celrep.2021.109504 PMC8299183

[66] Smolen KK, Plotkin AL, Shannon CP, Idoko OT, Pak J, Darboe A, Ontogeny of plasma cytokine and chemokine concentrations across the first week of human life. Cytokine 2021;148:155704. 34597920 10.1016/j.cyto.2021.155704 PMC8665647

[67] Kennedy RB, Oberg AL, Ovsyannikova IG, Haralambieva IH, Grill D, Poland GA. Transcriptomic profiles of high and low antibody responders to smallpox vaccine. Genes Immun 2013;14(5):277–85. 23594957 10.1038/gene.2013.14 PMC3723701

[68] IMPACC Manuscript Writing Team; IMPACC Network Steering Committee. Immunophenotyping assessment in a COVID-19 cohort (IMPACC): A prospective longitudinal study. Sci Immunol 2021;6(62):eabf3733. 10.1126/sciimmunol.abf3733. 34376480 PMC8713959

[69] Kennedy RB, Ovsyannikova IG, Haralambieva IH, Oberg AL, Zimmermann MT, Grill DE, Immunosenescence-related transcriptomic and immunologic changes in older individuals following influenza vaccination. Front Immunol 2016;7:450. 27853459 10.3389/fimmu.2016.00450 PMC5089977

[70] Bagwell CB, Hunsberger B, Hill B, Herbert D, Bray C, Selvanantham T, Multisite reproducibility of a human immunophenotyping assay in whole blood and peripheral blood mononuclear cells preparations using CyTOF technology coupled with Maxpar Pathsetter, an automated data analysis system. Cytometry B Clin Cytom 2020;98(2):146–60. 31758746 10.1002/cyto.b.21858 PMC7543682

[71] Hata Y, Hirono K, Yamaguchi Y, Ichida F, Oku Y, Nishida N. Minimal inflammatory foci of unknown etiology may be a tentative sign of early stage inherited cardiomyopathy. Mod Pathol 2019;32(9):1281–90. 31024045 10.1038/s41379-019-0274-0

[72] Belkaya S, Kontorovich AR, Byun M, Mulero-Navarro S, Bajolle F, Cobat A, Autosomal recessive cardiomyopathy presenting as acute myocarditis. J Am Coll Cardiol 2017;69(13):1653–65. 28359509 10.1016/j.jacc.2017.01.043 PMC5551973

[73] Morrocchi E, van Haren S, Palma P, Levy O. Modeling human immune responses to vaccination in vitro. Trends Immunol 2024;45(1):32–47. 38135599 10.1016/j.it.2023.11.002 PMC11688643

[74] Thomas S, Pak J, Doss-Gollin S, Ryff K, Beijnen E, Pedersen GK, Human in vitro modeling identifies adjuvant combinations that unlock antigen cross-presentation and promote T-helper 1 development in newborns, adults and elders. J Mol Biol 2024;436(4):168446. 38242283 10.1016/j.jmb.2024.168446 PMC10922990

[75] Sanchez-Schmitz G, Stevens CR, Bettencourt IA, Flynn PJ, Schmitz-Abe K, Metser G, Microphysiologic human tissue constructs reproduce autologous age-specific BCG and HBV primary immunization in vitro. Front Immunol 2018;9:2634. 30524426 10.3389/fimmu.2018.02634 PMC6256288

[76] Doss-Gollin S, Thomas S, Brook B, Abedi K, Lebas C, Auderset F, Human in vitro modeling of adjuvant formulations demonstrates enhancement of immune responses to SARS-CoV-2 antigen. NPJ Vaccines 2023;8(1):163. 37884538 10.1038/s41541-023-00759-y PMC10603059

[77] Lee AH, Shannon CP, Amenyogbe N, Bennike TB, Diray-Arce J, Idoko OT, Dynamic molecular changes during the first week of human life follow a robust developmental trajectory. Nat Commun 2019;10(1):1092. 30862783 10.1038/s41467-019-08794-x PMC6414553

[78] Zushin P-JH, Mukherjee S, Wu JC. FDA modernization act 2.0: transitioning beyond animal models with human cells, organoids, and AI/ML-based approaches. J Clin Invest 2023;133(21). 10.1172/JCI175824 PMC10617761 37909337

[79] Van Haren SD, Pedersen GK, Kumar A, Ruckwardt TJ, Moin S, Moore IN, CAF08 adjuvant enables single dose protection against respiratory syncytial virus infection in murine newborns. Nat Commun 2022;13(1). 10.1038/s41467-022-31709-2 PMC9346114 35918315

[80] Martino D, Schultz N, Kaur R, Van Haren SD, Kresoje N, Hoch A, Respiratory infection- and asthma-prone, low vaccine responder children demonstrate distinct mononuclear cell DNA methylation pathways. Clin Epigenetics 2024;16(1). 10.1186/s13148-024-01703-0 PMC11223352 38961479

[81] Vignolo SM, Diray-Arce J, McEnaney K, Rao S, Shannon CP, Idoko OT, A cloud-based bioinformatic analytic infrastructure and data management Core for the expanded program on immunization consortium. J Clin Transl Sci 2020;5(1):e52. 33948273 10.1017/cts.2020.546 PMC8057481

[82] Tryka KA, Hao L, Sturcke A, Jin Y, Wang ZY, Ziyabari L, NCBI’S database of genotypes and phenotypes: dbGaP. Nucleic Acids Res 2014;42(Database issue):D975–9. 24297256 10.1093/nar/gkt1211 PMC3965052

[83] Bhattacharya S, Dunn P, Thomas CG, Smith B, Schaefer H, Chen J, ImmPort, toward repurposing of open access immunological assay data for translational and clinical research. Sci Data 2018;5:180015. 29485622 10.1038/sdata.2018.15 PMC5827693

[84] Argelaguet R, Velten B, Arnol D, Dietrich S, Zenz T, Marioni JC, Multi-omics factor analysis-a framework for unsupervised integration of multi-omics data sets. Mol Syst Biol 2018;14(6):e8124. 29925568 10.15252/msb.20178124 PMC6010767

[85] Singh A, Shannon CP, Gautier B, Rohart F, Vacher M, Tebbutt SJ, DIABLO: an integrative approach for identifying key molecular drivers from multi-omics assays. Bioinformatics 2019;35(17):3055–62. 30657866 10.1093/bioinformatics/bty1054 PMC6735831

