# Supplementary material for: Longitudinal Meta-cohort study protocol using systems biology to identify vaccine safety biomarkers
Source: Vaccine. Author manuscript; Available in PMC 2026 Jul 22. (PMC13388017; doi:10.1016/j.vaccine.2025.127504)
Supplement: Suppl file 2 [file NIHMS2176053-supplement-Suppl_file_2.pdf]

PBMC Enhanced Panel for mass cytometry analysis

No.	Name	Species	Clone	Metal	Target
1	Anti-Human CD16 (3G8)-148Nd—100 Tests	Human	3G8	148Nd	CD16
2	Anti-Human CD8 (SK1)-168Er—100 Tests	Human	SK1	168Er	CD8a
3	Anti-Human CD20 (2H7)-171Yb—100 Tests	Human	2H7	171Yb	CD20
4	Anti-Human CD4 (SK3)-174Yb—100 Tests	Human	SK3	174Yb	CD4
5	Anti-Human CD45 (HI30)-Y89—100 Tests	Human	HI30	089Y	CD45
6	Anti-Human CD19 (HIB19)-142Nd—100 Tests	Human	HIB19	142Nd	CD19
7	Anti-Human IgD (IA6-2)-146Nd—100 Tests	Human	IA6-2	146Nd	IgD
8	Anti-Human CD11c (Bu15)-147Sm—100 Tests	Human	Bu15	147Sm	CD11c
9	Anti-Human TCRgd (11F2)-152Sm—100 Tests	Human	11F2	152Sm	TCRgd
10	Anti-Human CD3 (UCHT1)-154Sm—100 Tests	Human	UCHT1	154Sm	CD3
11	Anti-Human CD45RA (HI100)-155Gd—100 Tests	Human	HI100	155Gd	CD45RA
12	Anti-Human CD27 (L128)-158Gd—100 Tests	Human	L128	158Gd	CD27
13	Anti-Human HLA-DR (L243)-173Yb—100 Tests	Human	L243	173Yb	HLA-DR
14	Anti-Human CD196/CCR6 (G034E3)-141Pr—50 Tests	Human	G034E3	141Pr	CD196/CCR6
15	Anti-Human CD127/IL-7Ra (A019D5)-143Nd—100 Tests	Human	A019D5	143Nd	CD127/IL-7Ra
16	Anti-Human CD38 (HIT2)-144Nd—100 Tests	Human	HIT2	144Nd	CD38
17	Anti-Human CD194/CCR4 (205410)-149Sm—50 Tests	Human	L291h4	149Sm	CD194/CCR4
18	Anti-Human CD123/IL-3R (6H6)-151Eu—100 Tests	Human	6H6	151Eu	CD123/IL-3R
19	Anti-Human CD185/CXCR5 (RF8B2)-153Eu—100 Tests	Human	RF8B2	153Eu	CD185/CXCR5
20	Anti-Human CD28 (CD28.2)-160Gd—100 Tests	Human	CD28.2	160Gd	CD28
21	Anti-Human CD183/CXCR3 (G025H7)-163Dy—100 Tests	Human	G025H7	163Dy	CD183/CXCR3
22	Anti-Human CD45RO (UCHL1)-165Ho—100 Tests	Human	UCHL1	165Ho	CD45RO
23	Anti-Human CD24 (ML5)-166Er—100 Tests	Human	ML5	166Er	CD24
24	Anti-Human CD197/CCR7 (G043H7)-167Er—50 Tests	Human	G043H7	167Er	CD197/CCR7
25	Anti-Human CD25 (2A3)-169Tm—100 Tests	Human	2A3	169Tm	CD25/IL-2R
26	Anti-Human CD14 (M5E2)-175Lu—100 Tests	Human	M5E2	175Lu	CD14
27	Anti-Human CD56 (NCAM16.2)-176Yb—100 Tests	Human	NCAM16.2	176Yb	CD56/NCAM
28	Anti-Human CD66b (80H3)-162Dy—100 Tests	Human	80H3	162Dy	CD66b
29	Anti-Human CD161 (HP-3G10)-164Dy—100 Tests	Human	HP-3G10	164Dy	CD161
30	Anti-Human CD223/LAG-3 (11C3C65)-150Nd—100 Tests	Human	11C3C65	150Nd	LAG-3
31	Anti-Human CD274/PD-L1 (29E.2A3)-156Gd—100 Tests	Human	29E.2A3	156Gd	PD-L1
32	Anti-Human CD152/CTLA-4 (14D3)-170Er—100 Tests	Human	14D3	170Er	CTLA-4
33	Anti-Human CX3CR1 (2A9-1)-172Yb—100 Tests	Human	2A9-1	172Yb	CX3CR1
34	Anti-Human CD11a (HI111)-145Nd-Custom	Human	HI111	145Nd	CD11a
35	Anti-Human CD366/Tim-3 (F38-2E2)-159Tb-Custom	Human	F38-2E2	159Tb	Tim-3
36	Anti-Human CD279/PD-1 (EH12.2H7)-161Dy-Custom	Human	EH12.2H7	161Dy	PD-1
