# Supplementary material for: Longitudinal Meta-cohort study protocol using systems biology to identify vaccine safety biomarkers
Source: Vaccine. Author manuscript; Available in PMC 2026 Jul 22. (PMC13388017; doi:10.1016/j.vaccine.2025.127504)
Supplement: Suppl file 1 [file NIHMS2176053-supplement-Suppl_file_1.pdf]

INSIS Multi-OMICs Standard Operating Procedure (SOP)

Version 1.0 2024-08-05
Compiled by Ana Chang, M.S, Mahitha Donthireddy, M.A, and Joann Diray-Arce, PhD
Reviewed by Ofer Levy MD, PhD
Precision Vaccines Program, Boston Children's Hospital
INSIS Leads: Karina Top, MD, MS University of Alberta and Robert Chen, MD Brighton Collaboration

1. Protocol Synopsis 2
   1.0. Protocol Summary 2
   1.1. Current protocol number and approval date 2
2. Study Members and Governance Structure 3
   2.1 Member List and Roles 3
   2.2 INSIS Governance Structure: 6
3. Work Package Objectives 6
4. Training Plan 8
5. Study Procedures 8
6. Screening and Enrollment 9
   6.1 Cases 9
   6.2 Healthy Controls 9
   6.3 Controls with non-vaccine associated disease 10
   6.4 Case definitions: 10
   6.5 Recruitment and Consent 10
7. Biological Sample Collection and Processing 11
   7.1 Retrospective Sample Aliquoting Procedure 12
   7.2 Prospective Sample Collection: (only selected sites) 13
8. Biological Sample Shipping 19
9. Systems Biology Assays 21
   9.1 Serum sample assays 21
   9.2 PBMC processing 28
   9.3 RNA Sample Processing 31
   9.4 Assays planned for plasma samples 33
   9.5 Saliva sample processing 39

1. Protocol Synopsis

1.0. Protocol Summary

The International Network of Special Immunization Services (**INSIS**) aims to combine clinical and systems biology "*adversomics*" approaches to investigate rare Adverse Events of Special Interest (**AESIs**) detected after the introduction of vaccines for emerging diseases. Adversomics employs powerful technologies that comprehensively measure the inventory of molecules (e.g., DNA, RNAs, proteins, and metabolites) in a given sample. This approach has the major advantage of not making any assumptions regarding potentially relevant mechanisms but rather casting a broad/comprehensive net to define molecular pathways that are associated with and contribute to AESIs. Together these approaches will enable the characterization of the clinical spectrum, risk factors, and underlying mechanisms of postvaccination AESIs. This work will identify molecular signatures and pathways associated with AESIs that can be monitored in preclinical and clinical trials to estimate the potential of new vaccines to cause AESIs before deployment and inform regulatory assessment and immunization recommendations.

1.0.1. Objectives

1. To inform safety evaluation and risk-benefit assessment of new vaccines for emerging pathogens in low- and middle-income countries (**LMICs**) and high-income countries (**HICs**) through partnering with experts in LMICs to develop processes and capacity for case finding and evaluation of patients with post-vaccination AESIs, starting with myocarditis, pericarditis, and thrombosis with thrombocytopenia syndrome/vaccine-induced thrombocytopenia and thrombosis (**TTS/VITT**) and; developing and implementing case definitions and protocols for data and sample collection of post-vaccination AESIs and identify risk factors for AESIs associated with mRNA vaccines (i.e., myocarditis and pericarditis) and adenoviral vector vaccines (i.e., TTS/VITT).

2. Through genomics analysis of samples from AESI cases and controls, identify genetic variants strongly associated with an increased risk of myocarditis, pericarditis, and TTS/VITT following COVID-19 vaccination.

3. Through multi-OMIC studies, address knowledge gaps of the mechanistic basis of myocarditis, pericarditis, and TTS/VITT, their relationship to specific vaccine products and platforms, and populations at risk, to enhance safety assessment before emergency authorization and inform vaccine use in outbreak settings.

1.1. Current protocol number and approval date

INSIS Data Core Protocol Number: IRB-P00042327
IRB Exemption date: 27 January 2023

2. Study Members and Governance Structure

2.1 Member List and Roles

INSIS is hosted by University of Alberta, Canada and run by an INSIS Steering Committee formed by lead investigators from each member organization, WHO SAGE representative, and working group leads supported by a Program Manager.

Table 1. Member List and Roles

Group/Institution	Focus Area	Specific Activities
Canadian Special Immunization Clinic (SIC) Network, University of Alberta (Top)	Lead site and data center for SIC Network, experience leading international clinical trials and national research networks	• Overall network coordination, project management, subsite agreements<br>• Communications<br>• Recruitment of participants in Canada<br>• Oversight of clinical data analysis<br>• Oversight of knowledge dissemination
Brighton Collaboration (Chen)	Consortium of vaccine safety experts that sets international standard for AESI case definitions and data collection	• Inform network priorities<br>• Scientific coordination, international outreach<br>• Collaboration with Safety Platform for Emergency vACcines (SPEAC) activities, LMICs<br>• Contribute to data/sample collection protocols
Precision Vaccines Program (PVP), Boston Children's Hospital (Levy, Ozonoff, Arce; Proteomics: Steen)	Systems biology of human vaccine responses *in vivo* and *in vitro* to assess biomarkers predictive of vaccine safety and efficacy. Clinical & Data Coordinating Center for Human Immunology Project Consortium (HIPC) and national IMPACC COVID-19 immunophenotyping study. Human systems biology and *in vitro* modelling of vaccine induced biomarkers predictive of safety and efficacy.	• Data management and analysis<br>• Data and Material Transfer Agreements<br>• Broad specimen tracking system<br>• Oversight of data integration<br>• Human *in vitro* modeling of vaccine induced biomarkers<br>• Multi-OMIC analyses, including proteomics and metabolomics.<br>• Co-lead knowledge dissemination efforts
Adverse Events Following Immunisation Clinical Assessment Network (AEFI-CAN) (Crawford)	Consortium of Australia's SIC and Health Departments with scope for national research	• Contribute to data/sample collection and analysis protocols<br>• Recruitment of participants in Australia<br>• Contribute to data analysis, knowledge dissemination

Vanderbilt Vaccine Research Program, Vanderbilt University Medical Center (Creech)	Expert in vaccine evaluation, clinical case evaluation, investigation of AESIs	• Contribute to data/sample collection protocols<br>• Contribute to participant recruitment<br>• Contribute to data analysis, knowledge dissemination
Canadian Pharmacogenomics Network for Drug Safety (CPNDS), BC Children's Hospital Research Institute (Carleton)	Genomic analysis of adverse events, phenotyping of AESI, Adverse Events Following Immunization (AEFIs), selection of key Adverse Events (AEs) for genomic investigation	• Contribute to data/sample collection and analysis protocols<br>• Coordinate collaboration between INSIS and GVDN on sample collection and analysis<br>• Contribute to data analysis, interpretation, knowledge dissemination
Mayo Vaccine Research Group (VRG), Mayo Clinic (Kennedy, Poland)	Experts in transcriptomics, immunogenetic analysis, and humoral and cellular immune response markers	• Contribute to data/sample collection and analysis protocols<br>• RNASeq and immunologic analysis<br>• Contribute to/lead knowledge dissemination efforts
Global Healthcare Consulting (Kochhar)	Expert in vaccine safety research, clinical trials, strengthening pharmacovigilance systems in HICs and LMICs, and global vaccine policy. Serves on WHO Strategic Advisory Group of Experts on Immunization (SAGE), WHO Global Advisory Committee on Vaccine Safety (GACVS) WG on COVID-19 Vaccines Safety Preparedness, WHO Expert Steering Committee on Safety Surveillance in Pregnancy in LMICs and Brighton Collaboration Science Board AEFI guidance, protocols and for implementation in HICs and LMICs, and Brighton Collaboration Science Board	• Inform network priorities<br>• Contribute to data/sample collection protocols, international outreach and implementation in LMICs<br>• Contribute to interpretation of results and knowledge dissemination activities
Global Vaccine Data Network (GVDN) (Black/ Petoussis-Harris)	Experts in use of health data to evaluate vaccine safety and effectiveness and in genomics analysis of AESIs	• Keep INSIS members informed on complementary GVDN vaccine safety studies<br>• Contribute to data/sample collection protocols<br>• International outreach<br>• Implementation in LMICs

		• Contribute to interpretation of results and knowledge dissemination activities and other activities as outlined in the INSIS-GVDN MOU
African Leadership in Vaccinology Expertise (ALIVE) Network, Vaccines and Infectious Diseases Analytic Unit (VIDA), Divisions of Wits Health Consortium (Pty) Ltd (Cutland/Dangor)	Experts in vaccine safety research and clinical trials in the African region	• Contribute to data/sample collection protocols<br>• Recruitment of participants in the African region<br>• Contribute to data analysis, interpretation, knowledge exchange and dissemination
Ospedale Pediatrico Bambino Gesù (OPBG), Clinical Immunology and Vaccinology Unit (Palma)	Experts in multi-OMICs and extensive experience in clinical, basic and translational research on both primary and acquired immunodeficiencies. Primary vaccination center for patients affected by chronic diseases and for vaccine related adverse events	• Contribute to data/sample collection and clinical protocols<br>• Multi-OMICs and immunologic analysis<br>• Recruitment of participants<br>• Contribute to dissemination efforts
Ottawa Heart Institute (Liu)		• Contribute to data/sample collection protocols<br>• Recruitment of participants<br>• Contribute to dissemination efforts
Monash University (Tran)		• Contribute to data/sample collection protocols<br>• Recruitment of participants<br>• Contribute to dissemination efforts
Sydney Children's Health Network (Wood) University of Sydney (Chen)		• Contribute to data/sample collection protocols<br>• Recruitment of participants<br>• Contribute to dissemination efforts
McMaster University (Nazy)		• Contribute to data/sample collection protocols<br>• Recruitment of participants<br>• Contribute to dissemination efforts

Hospital for Sick Children-Pediatric Network (Yeung)		• Contribute to data/sample collection protocols<br>• Recruitment of participants<br>• Contribute to dissemination efforts
MGH (Yonker)		• Contribute to data/sample collection protocols<br>• Recruitment of participants<br>• Contribute to dissemination efforts
BWH (Lasky-Su)		• Contribute to data protocols<br>• Multi-OMICs and immunologic analysis<br>• Contribute to/lead knowledge dissemination efforts

2.2 INSIS Governance Structure:

The Steering Committee consists of the Nominate Principal Investigator (NPI), Co-lead from the Brighton Collaboration, representatives of the clinical networks in Australia, South Africa, and US, representatives involved in WHO SAGE, and systems biology and pharmacogenomics leads at the Boston Children's Hospital *Precision Vaccines Program*, Mayo Vaccine Research Group, BC Children's Hospital Research Institute, African Leadership in Vaccinology Expertise (**ALIVE**) Network, and *Ospedale Pediatrico Bambino Gesù* (**OPBG**). The Network Management Office (**NMO**) is located at University of Alberta and involves a Network Program Manager, Administrative staff, working with Communications, Contracts, and Finance personnel overseen by the NPI and Brighton Co-lead. The NMO interacts with the Task Force for Global Health, host of the Brighton Collaboration. An arms-length Scientific Advisory Board (**SAB**) will include experts in vaccine safety and systems biology as well as stakeholder representatives from funding organizations, regulators, public health, and LMICs.

3. Work Package Objectives

Work Package 1 (WP1): Establish Formal and Comprehensive Agreements within INSIS, and Finalize the INSIS-CEPI Multi-OMICs Strategy
Description: The INSIS OMICs Working Group and CEPI Translational Immunology team will co-develop a Multi-OMICs Strategy that will inform the approach to analyses of samples from myocarditis/pericarditis and TTS/VITT cases and controls. INSIS will complete the database build, finalize subsite agreements, data and materials transfer agreements, IRB/REB approvals, develop a catalogue of available samples at INSIS partner sites (biobanked and via ongoing recruitment), and initiate site recruitment.

The SPEAC Digital Transformations (**DT**) team will collaborate with INSIS to conduct user requirement assessment and provide specifications for INSIS DT activities, including development of an "adversomics" data catalogue. The DT team will also review data exchange protocols to support

adoption of SPEAC standards and tools within INSIS. The DT team will work with INSIS to metadata tag the INSIS database for entry into the SPEAC Vaccine Safety database inventory to support search by stakeholders for sources of case reports and specimens, that may already have been collected by other programs.

Objectives:

1. To formalize INSIS as a standing global network for vaccine safety with network agreements and SOPs, and ready INSIS to initiate multi-national studies;
2. To finalize the INSIS database and technical architecture for an "adversomics" data catalogue in collaboration with the SPEAC DT team and;
3. To engage with CEPI in finalizing a multi-OMICs analysis strategy for myocarditis, pericarditis, and TTS/VITT following SARS-CoV-2 vaccination.

Work Package 2 (WP2): Scale Multi-OMICs Approach In Vitro and In Vivo

Description: In WP2, sites will ship available samples on AESI cases and controls to INSIS labs. INSIS labs will begin running multi-OMICs assays as decided in the INSIS-CEPI Multi-OMICs Strategy and informed by the catalogue of available samples. Enrollment of AESI cases and controls will be completed by the end of WP2. Clinical data analysis will be conducted comparing characteristics of cases and controls. Preliminary genomics and OMICs data will be generated and integration of clinical data with OMICs will be initiated. Reports of clinical data analysis, preliminary results of genomics analysis led by the Global Vaccine Data Network's Dr. Carleton in collaboration with INSIS and OMICs data generation will be prepared. The INSIS OMICs Working Group will collaborate with the CEPI Translational Immunology team to review the findings and develop the approach to complete the final OMICs analysis. INSIS will continue to engage with LMIC partners (following completion of the 2-year pilot in ALIVE network sites supported by Dr. Top's CIHR-CEPI Award) to expand recruitment of AESI cases and controls in additional LMIC sites. INSIS members will also prepare and submit funding applications for co-funding of further multi-OMICs and genomics analysis of myocarditis, pericarditis, and TTS.

The SPEAC DT team will continue to work with INSIS to support adoption of SPEAC standards and tools within INSIS, to metadata tag INSIS database(s) as data are generated, and build the vaccine safety portal-based catalogue for data and information products in INSIS to support future access by outside partners (with appropriate permissions and approvals).

Objectives:

1. To determine clinical risk factors for myocarditis, pericarditis, and TTS following SARS-CoV-2 vaccination;
2. To generate preliminary data on genomic variants and multi-OMIC signatures that may be associated with myocarditis, pericarditis, and/or TTS following SARS-CoV-2 vaccination and;
3. To continue to engage LMIC partners to enhance capacity for AESI investigation and inclusion of individuals with AESIs in adversomics studies.

Work Package 3 (WP3): Complete Comprehensive OMIC Assays and Data Analysis to Identify AESI Biomarkers

Description: Upon meeting stage gate 2 and following CEPI governance approval will be determined based on final analysis approach for remaining samples (from ~ 100 additional myocarditis/pericarditis and TTS cases and ~ 300 controls) and co-funding availability. These additional samples will be used as validation cohorts to confirm findings of preliminary analyses and to increase power to assess differences in immunologic and molecular signatures between vaccine products, platforms, and demographic groups. Progress and co-funding will be reviewed in April 2026 and CEPI budget may be adjusted based on additional funding received in interim.

Objectives:

1. To identify genomic variants strongly associated with myocarditis, pericarditis, and TTS after SARS-CoV-2 vaccination.
2. To identify molecular and cellular signatures and predictive biomarkers of myocarditis, pericarditis, and TTS after SARS-CoV-2 vaccination.

4. Training Plan

- Training of study staff, including investigators, will be conducted prior to first enrollment. Initial training webinar sessions will be conducted to review Study Protocol, Clinical Database (REDCap), Sample Tracking Database (LDMS), Sample Processing Procedures, and Shipping Procedures Overview. Attendance will be documented as proof of training.
- Recordings of the initial training webinars will be made available on the study web portal.
- Protocol Signature pages will be collected from each Site-Investigator to document understanding of study procedures.
- Ongoing training and support will be provided during regularly scheduled meetings.

5. Study Procedures

Clinical assessment procedures of cases with myocarditis, pericarditis and TTS/VITT (e.g., investigation for other causes of myocarditis/thrombosis, diagnostic imaging) and other AESIs will be conducted as per routine clinical care. Investigations and additional referrals will be completed as indicated, according to local/national policies and procedures and physician discretion. INSIS investigators will harmonize clinical assessments to the extent possible.

Healthy controls will be matched approximately 3:1 to cases for multi-OMICs analysis by age group, sex, and vaccine type, and ancestry (where possible) or country. Controls with non-vaccine associated conditions will be frequency-matched to cases by age and sex. Serial blood samples will be obtained on controls for multi-OMICs pre-vaccination (where possible) and post-vaccination as shown in Figure 1.

Data collection:

Common data elements will be captured on all participants including age, ancestry (parents' country of origin), sex, gender (where available), recent SARS-CoV-2 infection, SARS-CoV-2 vaccination details, relevant past medical history, details of the AESI/condition including interval from vaccination to symptom onset (if applicable), Brighton Level of Certainty (**LOC**) (for AESI cases), level of medical care required, treatment, and outcome. At each follow up visit, data will be collected on any signs or symptoms including those associated with myocarditis, pericarditis or TTS/VITT to

detect persistent or recurrent disease activity. Data will be entered into a *Research Electronic Data Capture* (**REDCap**, v13.1.37) INSIS database.

6. Screening and Enrollment

6.1 Cases

Adults and children referred to an INSIS partner site for assessment of TTS/VITT or myocarditis or pericarditis or another AESI as a vaccine safety signal following SARS-CoV-2 vaccination will be invited to participate if they meet the following criteria:

Inclusion criteria:

- Age: Children who are age-eligible for SARS-CoV-2 vaccination in their country of residence and adults ≥18 years of age
- Received SARS-CoV-2 vaccination through a national or regional vaccination program
  AND
- Received a diagnosis of myocarditis, myopericarditis or pericarditis within 30 days of SARS-CoV-2 vaccination that meets the Brighton Collaboration Case Definition for myocarditis or pericarditis (Levels 1 and 2 of certainty)
  OR
- Received a diagnosis of TTS/VITT within 30 days of SARS-CoV-2 vaccination that meets the Brighton Collaboration Case Definition for TTS (Levels 1 and 2 of certainty)
- Participant or caregiver (if <18 years) able to provide informed consent

Exclusion criteria:

- Confirmed other cause for myocarditis/pericarditis and TTS/VITT, besides SARS-CoV-2 vaccination

6.2 Healthy Controls

Inclusion criteria:

- Age: Children who are age-eligible for SARS-CoV-2 vaccination in their country of residence and adults ≥18 years of age
- Received SARS-CoV-2 vaccination through national or regional vaccination program
  AND
- Did not develop signs or symptoms of myocarditis or pericarditis or TTS/VITT following SARS-CoV-2 vaccination
  AND
- Require further dose(s) of a SARS-CoV-2 vaccine (e.g., 2nd or booster dose) OR have stored samples from a previous SARS-CoV-2 vaccination OR received SARS-CoV-2 vaccination within prior 30 days
- Participant or caregiver (if <18 years) able to provide informed consent

Exclusion criteria:

- Serious AE following SARS-CoV-2 vaccination (i.e., requiring hospitalization, life-threatening, resulting in permanent disability or death)
- History of previous myocarditis, pericarditis, or inflammatory cardiac disease

- History of coagulation or bleeding disorder or thrombocytopenia

6.3 Controls with non-vaccine associated disease

Inclusion criteria:

- Age: Children who are age-eligible for SARS-CoV-2 vaccination in their country of residence and adults ≥18 years of age
- Diagnosis of myocarditis or pericarditis or TTS-like condition (e.g., heparin-induced thrombocytosis (**HIT**)) in past 7 days NOT associated with SARS-CoV-2 vaccination AND consents to blood collection
  OR
- History of myocarditis or pericarditis or TTS-like condition (e.g., heparin-induced thrombocytosis (**HIT**)) NOT associated with SARS-CoV-2 vaccination AND had blood samples collected and biobanked at time of initial presentation
- Participant or caregiver (if <18 years) able to provide informed consent

Exclusion criteria:

- History of serious AE following SARS-CoV-2 vaccination

6.4 Case definitions:

TTS/VITT: Brighton Collaboration case definitions for TTS/VITT (revised 2024) will be applied to post-vaccine cases and non-vaccine associated cases (applicable to TTS only). Cases of HIT or similar conditions that meet some or all criteria of the TTS definition (other than lack of heparin exposure) will be eligible for inclusion as non-vaccine associated TTS/VITT controls. TTS/VITT cases will be analyzed in two groups: VITT and TTS.

Myocarditis/pericarditis: Brighton Collaboration case definitions for myocarditis and pericarditis will be applied to post-vaccine and non-vaccine associated cases. These events will be analyzed in 2 groups: myocarditis (with or without signs of pericarditis) and pericarditis without myocarditis.

Case definitions for additional AESIs that represent new or emerging vaccine safety signals will be based on Brighton Collaboration Case Definitions or on an agreed-upon internationally accepted case definition. Both levels 1 and 2 of certainty will be eligible.

6.5 Recruitment and Consent

Recruitment:

Cases and controls with non-vaccine associated disease will be recruited from among patients referred to INSIS-affiliated special immunization clinics and specialists for diagnosis, treatment and follow up of their condition, patients who had blood samples submitted for diagnostic testing at an INSIS-affiliated site (where approvals are in place to use residual sample for research), or via existing disease registries and biobanking studies.

Healthy controls will be enrolled from first degree relatives of index cases, participants in clinical trials, surveillance, and observational studies of SARS-CoV-2 vaccines who did not report AEFIs and indicated interest in being contacted for research and/or provided consent to use their data and samples for research in international studies, and participants in hospital research registries, as well as the public via social media.

Consent:
Written or electronic consent will be required for data and sample collection (or retrieval/use of biobanked samples), transfer of a limited de-identified dataset to the central REDCap database, and transfer of saliva and/or blood samples to INSIS biobanking sites and specialized laboratories for analysis. Participants will also consent to linkage of their biological data to their clinical data. Consent will be obtained by a member of the study team in accordance with local requirements. Participants who previously signed consent for biobanking and/or use of their data and samples will only be approached to re-consent if their previous consent does not cover their use as outlined in this protocol. Data and samples will only be transferred out of country once all necessary approvals and data and material transfer agreements (**MTAs**) are fully executed.

Consent to provide samples for genomics analysis (and transfer of clinical data) will be obtained from cases and controls under the GVDN genomic analysis protocol and consent.

If serial blood collection is not feasible or participants decline a blood draw, they may opt out of sampling for multi-OMICs analysis and provide only clinical data to the INSIS database and consent separately to participate in the GVDN genomics protocol, whereupon GVDN will be granted access to their clinical data in the INSIS database.

7. Biological Sample Collection and Processing

Overall Processing Pipeline

The diagram below outlines the assay prioritization workflow for the INSIS project. Biological sample collection and processing will be performed at INSIS Clinical and Lab sites.

Figure 1. INSIS Overall Processing Pipeline

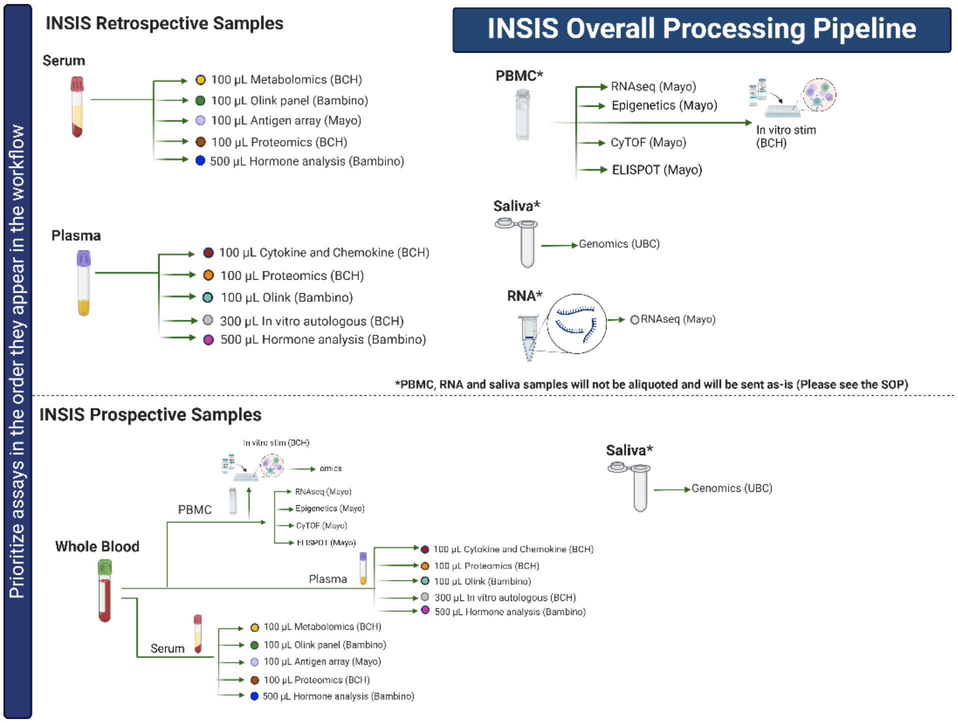

7.1 Retrospective Sample Aliquoting Procedure

Purpose: This protocol outlines the procedures for handling and aliquoting frozen plasma and serum samples stored at INSIS retrospective sites.

NOTE: Please DO NOT thaw and aliquot PBMC and RNA samples from retrospective samples. These samples will be shipped in their current state to the core facilities to avoid loss of viability due to multiple freeze-thaw cycles.

For the serum and plasma samples, avoid repeated freeze-thaw cycles. Coordinate with the PVP Team to schedule processing, thawing and shipping time. Please use LDMS to capture information and track newly aliquoted samples.

Saliva samples should be stored and shipped at ambient temperature.

Materials/Reagents for thawing plasma and serum samples:

- Frozen plasma, serum samples
- Personal Protective Equipment (**PPE**): lab coat, gloves and eye protection
- INSIS defined cryotubes
- Color-coded cryolabels
- Pipettes and sterile pipette tips
- Cryoboxes or racks for sample organization
- INSIS tube labels

Procedure for thawing plasma and serum samples:

1. Ensure all materials and equipment are clean and ready to use.

2. Label aliquot tubes with the INSIS defined label and other relevant information before starting the handling process. Fill out sample information using INSIS-specified LDMS tracking database.
3. Wear appropriate PPE throughout the procedures.
4. Retrieve the plasma and/or serum samples from the freezer and immediately transfer to a 2°C - 8°C refrigerator to thaw slowly (Guideline: 30-60 minutes/mL of liquid). Avoid room temperature or water bath thawing to minimize degradation in the samples.
5. Once plasma or serum samples have thawed, gently mix by inverting the tube 5-6 times to ensure homogeneity. Avoid vigorous shaking.
6. Using a pipette, aliquot the required volume of sample (refer to the visual summary of serum and plasma assays) into the pre-labeled tubes. Change pipette tips between samples to prevent cross-contamination.
7. Affix the corresponding colored cryo-stickers on top of the labeled tubes. (refer to Table 2).
8. Immediately store the aliquoted samples at the specified temperature until ready for shipping needed for assays. For long-term preservation, store aliquots at –80°C. Refer to Table 2 below for long-term storage guidelines, which typically recommend storage at –80°C.
9. Double-check and make sure you saved the information in the INSIS-specified LDMS tracking database.

Sample type	Prioritization of Assay	Assay	Cryo Label Color	Color Code	Volume needed
Serum	1	Metabolomics	Yellow	YE	100 µL
	2	Olink panel	Green Apple	GA	100 µL
	3	Antigen array	Lavender	LA	100 µL
	4	Proteomics	Brown	BR	100 µL
	5	Hormone analysis	Blue	BL	500 µL
Plasma	1	Cytokine and Chemokine	Red	RE	100 µL
	2	Proteomics	Orange	OR	100 µL
	3	Olink panel	Green seafoam	GS	100 µL
	4	In vitro autologous	Silver	SI	300 µL
	5	Hormone analysis	Hot pink	PI	500 µL

7.2 Prospective Sample Collection: (only selected sites)

Blood sample collection:
For cases and controls, at each timepoint (see Table 3), peripheral blood (5-15 ml) will be collected according to one of the protocols outlined below or an equivalent protocol. Volumes will depend on the exact tubes and sample protocol used. Stored blood samples may be used if appropriately cryopreserved. Where processing facilities are available, whole blood (~20-30 ml) will be collected

for peripheral blood mononuclear cell (**PBMC**) isolation. Additional sample types, including plasma and serum, will be collected according to site or other study-specific protocols.

DNA analysis: A saliva or blood sample suitable for DNA extraction and genotyping will be collected from cases and controls with consent according to the GVDN genomics protocol.

Samples for downstream Multi-OMIC assays will be stored at the participating site or shipped on dry ice to an INSIS laboratory with biobanking facilities, site where they will be stored at -80°C until analysis.

Table 3. Schedule of study procedures, prospectively enrolled cases and controls for multi-OMICs analysis. *

	**During adverse event/onset myocarditis/TTS/VITT**	**2-8 weeks after event onset†**	**~12-20 weeks after onset**	**~6-24 months after onset**
Cases and controls with non-vaccine associated disease	• Consent (if possible)<br>• Data collection<br>• Blood collection**	• Consent‡<br>• Data collection<br>• Blood collection	• Consent (if not yet obtained) ‡<br>• Data collection<br>• Blood collection	• Data collection<br>• Blood collection
	Pre-vaccination (if available)	~3-90 days post-vaccination**	~12-20 weeks post-vaccination	~6-24 months post-vaccination
Controls	• Consent (if possible)<br>• Data collection<br>• Blood collection**	• Consent‡<br>• Data collection<br>• Blood collection	• Consent (if not yet obtained) ‡<br>• Data collection<br>• Blood collection	• Data collection<br>• Blood collection

*Participants may be included if multi-OMICs samples are available from only 1 timepoint (e.g., during the acute adverse event) but 2 or more timepoints are preferred within these approximate timeframes (preferably an acute sample and follow up sample). In some cases an early follow up sample and late follow up sample (e.g., 3 and >6 months) will be acceptable. A maximum of 90 ml of blood will be drawn on children <16 years of age.
†At time of first specialist assessment (e.g., cardiology, special immunization clinic)
‡If not consented at presentation, consent will be obtained at time of specialist assessment for retrieval of residual serum/plasma from initial presentation.

**Aim for 3–7 days post-vaccination for controls matched to myocarditis cases; 5-42 days post-vaccination for controls matched to TTS/VITT cases, longer timeframes may be appropriate for additional AESI of interest.

7.2.1 Whole Blood sample processing for prospective samples

This protocol was adapted from IMPACC (DOI: 10.1126/sciimmunol.abf3733), Vanderbilt University Medical Center SOP, and EPIC HIPC Protocol (https://www.nature.com/articles/s41467-019-08794-x).

Sampling Schedule

Blood was collected in different tubes depending on the sites' protocol.

Whole blood assays visual summary

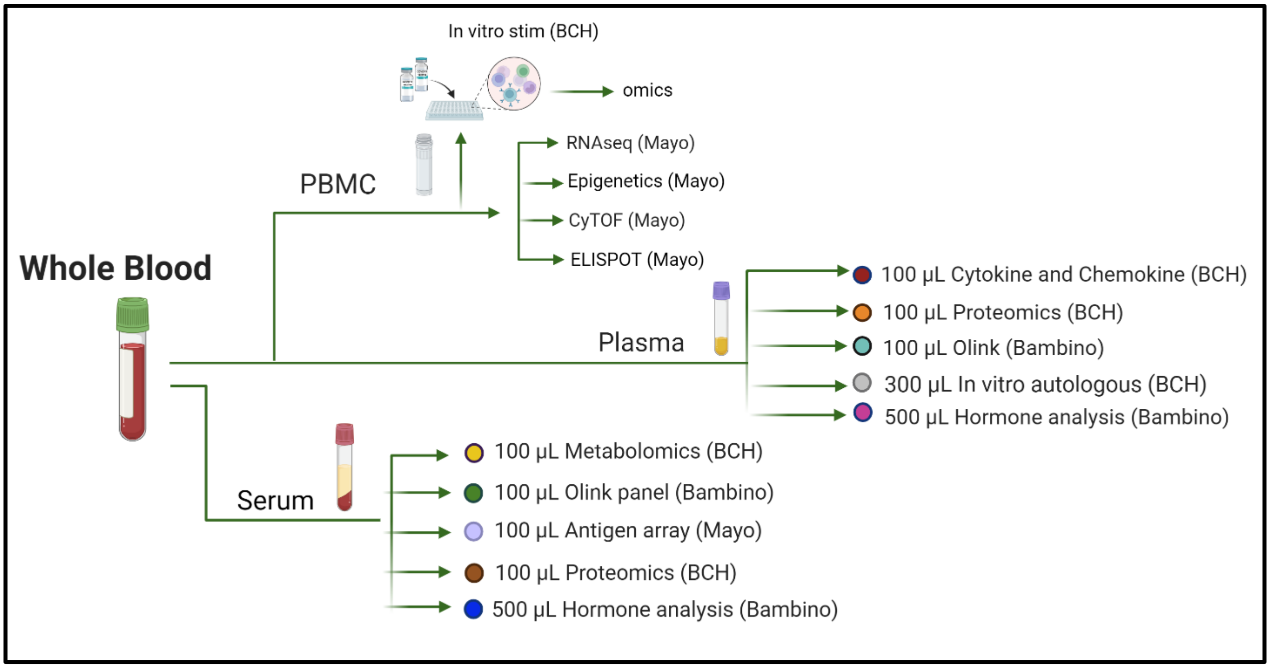

7.2.1.1 For serum sample processing:

Materials:

- Whole blood drawn in appropriate tube (without any anticoagulant, preferably *SST Greiner tube*)
- Tabletop centrifuge (*Beckman Coulter Allegra X-12R* or equivalent)

- INSIS-specified cryovials with colored labels
- Adjustable pipettor with tips
- -80°C freezer
- LDMS tracking database

Procedure:

1. Procedures should be done following universal precautions for blood borne pathogens and under a laminar flow hood or enclosed environments with the appropriate HEPA filters.
2. Whole blood drawn in a tube without anticoagulant (e.g. *SST Greiner tube*) should be left at room temperature after drawing for a minimum of 30 mins and maximum of 2 hours. The samples should then be placed at 4°C. Samples should be processed within 24 hours of collection.
3. Allow the serum tube to clot for at least 30 minutes at room temperature in a vertical position.
4. Prepare and label cryovials.
5. The tube should then be placed in a tabletop centrifuge and serum clarified by centrifugation at 2000 x g for 10 mins at 4°C. Brake can be applied on high. (Swinging out rotor and brake ON).
6. After centrifugation, the gel should be intact, and the cells and serum completely separated.
   a. Do not re-centrifuge the tube if the barrier is incomplete.
   b. Serum should be layered on top of cells and can be aliquoted into labeled cryovials with an adjustable pipettor.
7. Aliquot serum into cryovials and earmark each cryovial with the information below:
   Table 4. Serum Assay Prioritization

Sample type	Prioritization of Assay	Assay	Cryo Label Color	Color Code	Volume needed
Serum	1	Metabolomics	Yellow	YE	100 µL
	2	Olink panel	Green Apple	GA	100 µL
	3	Antigen array	Lavender	LA	100 µL
	4	Proteomics	Brown	BR	100 µL
	5	Hormone analysis	Blue	BL	500 µL

8. Serum can be stored at -80°C in labeled cryoboxes until shipment.

7.2.1.2 For PBMC processing:

Adapted from: Vanderbilt SOP PBMC 101.007

NOTE: Whole blood should be collected in a tube with an anticoagulant (preferably *EDTA*).

Please store an additional autologous plasma (300 µL) together with cryopreserved PBMC to be sent to PVP. Prior to freezing, plasma should be centrifuged at 3000 x g for 15 minutes to remove platelets.

Platelet-poor plasma (**PPP**) should be transferred to new vials. Two vials of 100 µL are sufficient for the in vitro assays as outlined below under the in vitro assay SOP.

Materials:

- EDTA preserved whole blood
- RPMI 1640
- *Ficoll Hypaque* (*Sigma Cat*# 1077-1 or equivalent)
- 50 ml conical tubes
- Tabletop centrifuge (Beckman Coulter Allegra-14R or equivalent)
- Pasteur pipettes
- Standard adjustable pipettors
- Serological pipettes (25, 10, 5 ml)
- cryotubes with labels
- Heat inactivated FBS
- DMSO
- Cool Cell or Mr Frosty
- Liquid nitrogen storage tank
- Microcentrifuge tube
- LDMS database

Procedure:

To Obtain Peripheral Blood Mononuclear Cells

1. When working with blood or blood products, standard OSHA blood borne pathogen precautions should be followed.
2. Procedure should be executed in a BSL-2 compliant laminar flow hood.
3. Each sample should be filled out completely and logged in INSIS-specific LDMS tracking. Prepare the cryotubes and affix the labels.
4. Samples can remain at room temperature for a maximum of 18 hours before processing. It is preferable to process within 4 hours when possible.
5. EDTA-preserved whole blood should be diluted with RPMI 1640 in a 50 ml conical tube in a ratio of approximately 15 ml of whole blood to 20 ml of RPMI 1640. For other blood volumes, an approximate 1:2 ratio should be maintained. Invert the tube gently to mix media and blood.
    a. If 5ml of less of whole blood is obtained, a 15 ml conical tube can be used with approximately 4ml of ficol for gradient.
6. Underlay 12 ml of *Ficoll Hypaque* to the media blood mixture with a 10 ml serological pipette being careful to not mix the layers.
7. *Ficoll* should be at room temperature to ensure appropriate density.
8. In a tabletop centrifuge, spin 50 ml conical tubes at 2500 rpm (1455g) for 25 mins.
9. Remove tubes from centrifuge and place in a rack under hood.
10. Remove media layer, using a 25 ml pipette, leaving approximately 4 ml of media over the buffy coat.
11. Remove the remaining 4 ml of media layer with buffy coat, using a Pasteur pipette or serological pipette, aspirating as little *Ficoll* as possible.

12. Place buffy coat cells into a separate 50 ml conical tube. Cells from three buffy coats can be placed into one 50 ml conical tube.
13. Fill 50 ml conical tube with RPMI 1640 to 50 ml.
14. In a tabletop centrifuge, spin cells at 1800 rpm (754g) for 18 mins.
15. Remove from centrifuge and decant media into discard container without disturbing the pellet.
16. Resuspend cells in 1 ml of RPMI 1640.
17. Remove 10 µl of cell suspension from 50 ml conical tube and place it in a microcentrifuge tube containing 390 µl of 0.04% Trypan blue.
18. Dilution can be adjusted as needed based on the anticipated number of cells.
19. Count cells on hemocytometer or other automated cell counter.
20. Add RPMI 1640 to fill tube with remaining cells.
21. In a tabletop centrifuge, spin cells at 1500 rpm (524g) for 15 minutes.
22. Make freeze media consisting of 90% FBS and 10% DMSO. Make sufficient amount for current processing batch. Make sterile, fresh media with each processing batch under a hood. Do not store mixed freeze media. Screened FBS indicates that the FBS lot was tested with freeze procedure to ensure cell viability upon thawing.
23. Remove from centrifuge and decant media into discard container.
24. Resuspend cells in freeze media to 5 x 10^6 cells/ml.
    a. If more than 150 x 10^6 cells are obtained, they should be frozen at a concentration of 10 x 10^6 cells/ml.
25. Immediately aliquot 1 ml of suspension into 1.8 ml labeled cryotubes for cryopreservation.
26. Double-check that the samples are logged into the INSIS LDMS Tracking database and that the labels are affixed properly.
27. Place cells in a pre-chilled (4ºC) isopropyl alcohol freeze chamber or Cool Cell and place at –80ºC for a minimum of 24 hours or a maximum of 2 weeks.
28. Long term storage for frozen cells in liquid nitrogen gas phase

7.2.1.3 For plasma processing:

Materials:

- 15 mL conical tubes or 50 mL conical tubes
- Disposable Transfer pipettes
- Pipette tips
- Remaining blood from EDTA-preserved tubes

Procedure:
1. Each sample should be filled out completely and logged in INSIS-specific LDMS tracking. Prepare the cryotubes and affix the labels.
2. Centrifuge remaining blood sample at 1000 x g for 10 min at RT with swinging-out rotor and brake OFF.
3. Aliquot plasma off the top cell layer in color-coded cryovials as noted in the table below.

Table 5. Plasma Assay Prioritization

Sample type	Prioritization of Assay	Assay	Cryo Label Color	Color Code	Volume needed
Plasma	1	Cytokine and Chemokine	Red	RE	100 µL
	2	Proteomics	Orange	OR	100 µL
	3	Olink panel	Green seafoam	GS	100 µL
	4	In vitro autologous	Silver	SI	300 µL
	5	Hormone analysis	Hot pink	PI	500 µL

4. Place the tubes in assigned boxes for each assay. Store at -80°C for shipment.

7.2.2 Prospective Saliva sample collection (GVDN protocol):

Procedure:

1. Saliva DNA collection kits will be prepared for individual participant use. Each kit contains one saliva DNA collection kit (*Oragene®*) with instructions for use along with a self-addressed, stamped return envelope with instructions for return. These kits can be mailed to participants along with a consent form and returned in a post-paid mailer.
2. The *Oragene®* kit is used to collect saliva from which DNA is extracted.
3. Saliva is obtained by asking the participant to spit into the saliva collection tube to the inscribed fill line.
4. Each kit will have a unique, participant-specific study ID. Henceforth, only the study ID is used to identify the participant.
5. Samples will then be stored and shipped at room temperature.
6. *Oragene®* collection kits are stable at room temperature for years because of proprietary reagents that prevent bacterial growth and minimize chemical hydrolysis of DNA.
7. Saliva samples will be stored as received and DNA extracted once samples are ready for genotyping and analysis.

8. Biological Sample Shipping

Table 6. Sample Shipment Summary

Sample type	Assay	Cryo Label Color	Color Code	Volume needed	Shipment Material	Initial shipping destination	Final destination Core lab*
Serum	Metabolomics	Yellow	YE	100 µL	Dry Ice	PVP/BCH	PVP/BCH
	Olink panel	Green Apple	GA	100 µL	Dry Ice	PVP/BCH	OPBG

	Antigen array	Lavender	LA	100 µL	Dry Ice	PVP/BCH	Mayo
	Proteomics	Brown	BR	100 µL	Dry Ice	PVP/BCH	PVP/BCH
	Hormone analysis	Blue	BL	500 µL	Dry Ice	PVP/BCH	OPBG
Plasma	Cytokine and Chemokine	Red	RE	100 µL	Dry Ice	PVP/BCH	PVP/BCH
	Proteomics	Orange	OR	100 µL	Dry Ice	PVP/BCH	PVP/BCH
	Olink panel	Green seafoam	GS	100 µL	Dry Ice	PVP/BCH	OPBG
	In vitro autologous	Silver	SI	300 µL	Dry Ice	PVP/BCH	PVP/BCH
	Hormone analysis	Hot pink	PI	500 µL	Dry Ice	PVP/BCH	OPBG
Whole Blood	In vitro assay	Beige	BG	Dry Ice	PVP/BCH	PVP/BCH	
PBMC	RNAseq			Liquid Nitrogen	Mayo	Mayo	
	CyTOF			Liquid Nitrogen	Mayo	Mayo	
	Epigenetics			Liquid Nitrogen	Mayo	Mayo	
	In vitro assay	Beige	BG	Liquid Nitrogen	Mayo	PVP/BCH	
Saliva	Genomics			Ambient	PVP/BCH	UBC	
RNA	RNAseq	White		Dry Ice	Mayo	Mayo	

*PVP/BCH will distribute the samples to the final destination core labs except for PBMC samples which will go directly to Mayo Clinic.

9. Systems Biology Assays

Note: The procedures below will be performed at the core assay sites by core lab members.

9.1 Serum sample assays

Serum assays visual summary

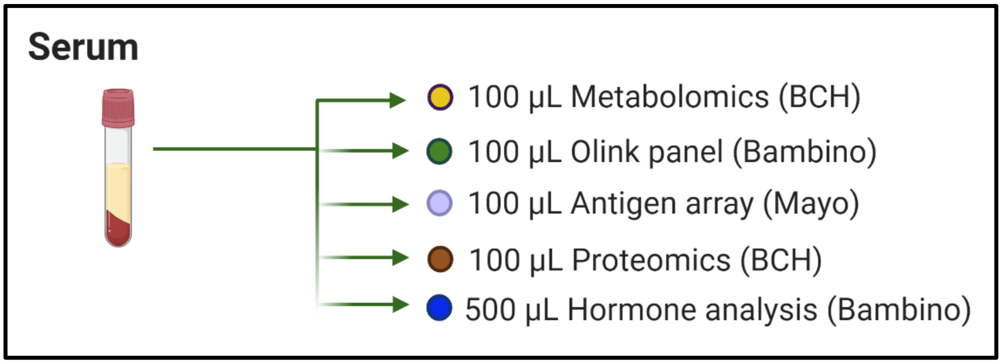

9.1.1 Metabolomics (BCH-PVP)

Goal: Metabolomics provides comprehensive assessment of the metabolome in relation to vaccine AEs.

Reagents and software:

- Vacutainer for serum
- Chilled, polypropylene tubes
- Liquid nitrogen
- *R* (https://www.r-project.org/)
- *Bioconductor* (https://www.bioconductor.org/)

Procedure:

1. Aliquot the serum sample into chilled, polypropylene tubes and flash-freeze in liquid nitrogen. Recommended volume is 50 µl serum.
2. Store samples at -80C until shipment to Metabolon (Durham, NC) which is retained by the *Precision Vaccines Program* on a fee-for-service basis.
3. Following receipt, enter the samples into the Metabolon LIMS system: Assign a unique identifier associated with the original source identifier only.
4. Prepare the samples using the automated *MicroLab STAR*® system from Hamilton Company
5. Add recovery standards prior to the first step in the extraction process for QC purposes.
6. To remove protein, dissociate small molecules bound to protein or trapped in the precipitated protein matrix, and to recover chemically diverse metabolites, proteins will be precipitated with

methanol under vigorous shaking for 2 min (*Glen Mills GenoGrinder* 2000) followed by centrifugation.

7. Divide the resulting extract into five fractions: two for analysis by two separate reverse phase (RP)/UPLC-MS/MS methods with positive ion mode electrospray ionization (**ESI**), one for analysis by RP/UPLC-MS/MS with negative ion mode ESI, one for analysis by HILIC/UPLC-MS/MS with negative ion mode ESI, and one sample was reserved for backup.
8. Place the samples briefly on a *TurboVap®* (Zymark) to remove the organic solvent. Store the sample extracts overnight under nitrogen before preparation for analysis.
9. Run the samples and analyze by liquid chromatography mass spectrometry

9.1.2 Proximity Extension Assay based proteomics (OLINK)

Sample types

Adapted from Olink® Target 96 User Manual.

Olink panels have been validated using EDTA plasma and serum samples. A range of additional sample types are compatible with the technology. For example, citrate plasma, heparin plasma, tissue and cell lysates, CSF and saliva. Different sample matrices are expected to affect the detection of specific proteins in different ways. In addition, extreme levels of IgG or fluorescent particles can interfere with the *Olink* assay.

For more information on sample types, please see the Data Validation documents corresponding to each panel or contact *Olink* support at support@olink.com.

Note: For prospective sample collection, EDTA plasma is the preferred storage medium over sodium citrate.

Pipettes: A multichannel pipette and a reverse pipetting technique must be used in the reagent transfer step. Maintain and calibrate the pipettes regularly.

Procedure:

Sample preparation

1. Use a 96-well PCR plate format, preferably with a full skirt. All plates need to be able to withstand -80° C, be dry-ice resistant and easily re-sealable.
2. Ensure that the samples are randomized, or in the order ready to be run and compatible with the plate layout.
3. Ensure that each well is separately sealed using an adhesive film or individual seals.
4. Clearly mark sample plates or tubes with a simple alphanumeric code that you can later identify (“A, B, C”, “1, 2, 3”, or “A1, A2, A3”) using temperature-resistant labels or marker pen.
5. Use unique sample identification names or numbers.

Sample dilution step for 1:10 panels

1. Thaw the Sample Diluent, vortex and empty the bottle into a multichannel pipette reservoir (minimum volume 15 mL).
2. Mark a 96-well plate as "Dilution Plate".

3. Pipette up and down in the Sample Diluent a few times in the sample diluent to pre-condition the pipette tips.
4. Transfer 9 µL of the Sample Diluent to each well of columns 1-11 and positions A-B in column 12 on the 96-well plate, using reverse pipetting. Pipette the Sample Diluent carefully to avoid foaming.
5. Vortex the sample plate using the *Eppendorf MixMate® Microplate Shaker* 30 seconds and spin down the liquid at 400-1000 x g, for 1 minute at room temperature. Carefully transfer 1 µL of your samples and pooled sample controls according to your plate layout to the Dilution Plate using forward pipetting.
6. Seal both the original sample plate and the Dilution Plate with adhesive plastic film.
7. Vortex the Dilution Plate thoroughly using the *MixMate®* for 30 seconds.
8. Spin down the content at 400-1000 x g for 1 minute at room temperature.
9. Double check that all wells in the plate contain the same volume. Note any deviations. This information is needed to interpret the data in the statistical analysis step.

Incubation step instruction

1. Thaw the samples.
2. Vortex the Negative Control, Inter-plate Control and Sample Control and spin briefly. Add 5 µL of the controls to an 8-well strip according to the following order:

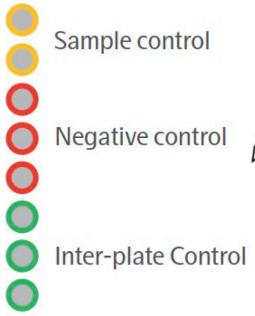

NOTE: *If running a diluted panel, the two sample controls will be placed on the sample plate and not in the control strip.*

3. Prepare the Incubation Mix in a microcentrifuge tube according to the table below. Vortex and spin each reagent before adding it to the mix.
4. Vortex and spin down the Incubation Mix. Transfer 47 µL of the Incubation Mix to each well of a new 8-well strip.
5. Pre-condition the pipette tips and transfer 3 µL of Incubation Mix to the bottom of the wells of a new 96-well plate by reverse pipetting and name it Incubation Plate. Use the same pipette tips for the entire plate. Pipette near the surface of the Incubation Mix to prevent liquid from sticking to the outside of the pipette tips.
6. Vortex the plate with samples using the *MixMate®* for 30 seconds and spin down the liquid at 400-1000 x g, for 1 minute at room temperature. If samples are in tubes, use a regular pipette to transfer 1 µL of each sample, using a multichannel pipette, to the bottom of the wells of the Incubation plate according to your sample plate layout.
7. Seal the Incubation Plate thoroughly with an adhesive plastic film, spin at 400-1000 x g for 1 minute at room temperature.
8. Double check that all wells in the plate contain the same volume. Note any deviations. This information is needed to interpret the data in the statistical analysis step.

9. Incubate the Incubation Plate overnight at +4 °C for 16-22 hours in a refrigerator or cold room where the light is off when the door is closed.

Extension and amplification step

In the morning of the following day, the extension and amplification steps take place. Unique DNA reporter sequences for each target protein are generated and pre-amplified using regular PCR.

1. Allow the PEA Solution to reach room temperature. Vortex and spin down briefly before use.
2. Pre-heat the PCR machine to 50 °C and pause the program.
3. Spin down the Incubation Plate at 400-1000 x g for 1 minute at room temperature.
4. Prepare the Extension Mix in a 15 mL tube according to the following table:

Extension Mix	Per 96-well plate (µL)
High Purity Water	9385
PEA Solution	1100
PEA Enzyme	55
PCR Polymerase	22
**Total**	10 562

5. Vortex the Extension Mix and pour it into a multichannel pipette reservoir.
6. Carefully remove the adhesive film from the Incubation Plate.
7. Start a 5-minute timer and transfer 96 µL of Extension Mix to the upper parts of each of the well walls of the Incubation Plate using reverse pipetting. Use the same pipette tips throughout the plate.
8. Seal the plate with a new adhesive plastic film.
9. Use the *MixMate®* to vortex the plate thoroughly for 30 seconds to ensure that all wells are mixed before spinning it down.
10. Double check that all wells in the plate contain the same volume. Note any deviations. This information is needed to interpret the data in the statistical analysis step.
11. Take the Incubation Plate to the PCR room.
12. Immediately place the Incubation Plate in the PCR instrument and resume the PEA program. The PEA program takes approximately 1 hour and 30 minutes. Refer to *Olink®* user manual section 6.2.1 extension and pre-amplification program for more information.
13. When the PCR PEA program is finished (~1 hour 55 minutes), continue to detection step.

Detection step

1. Remove the Extension products from the PCR instrument. Vortex and spin down the liquid.
2. Double check that all wells in the plate contain the same volume. Note any deviations. This information is needed to interpret the data in the statistical analysis step.
3. Thaw the Primer Plate and Detection Solution. Vortex and spin down the Detection Solution and Primer Plate. Keep the Detection Enzyme and PCR Polymerase in a freezing block or on ice.
4. Prepare the Detection Mix in a microcentrifuge tube.

Detection Mix	Per 96-well plate (µL)
Detection Solution	550.0
High purity water	230.0
Detection Enzyme	7.8
PCR Polymerase	3.1
**Total**	790.9

5. Vortex the Detection Mix and spin briefly. Transfer 95 µL of the mix to each well of an 8-well strip.
6. Use a multichannel pipette to transfer 7.2 µL of the Detection Mix to each well of a new 96-well plate by reverse pipetting. Use the same pipette tips throughout the plate. Name this plate, Sample Plate.
7. Carefully remove the adhesive film from the Incubation Plate.
8. Transfer 2.8 µL from the extension products in the Incubation Plate to the Sample Plate using a multichannel pipette and forward pipetting. Change tips between each column.
9. Seal both the Sample Plate and the plate with extension products with adhesive plastic film.
10. Vortex and spin the Sample Plate at 400-1000 x g, for 1 minute at room temperature together with the Primer Plate.
11. Double check that all wells in the Sample Plate contain the same volume. Note any deviations. This information is needed to interpret the data in the statistical analysis step.
12. Remove the primed IFC chip from the *Olink* Signature Q100.
13. Carefully remove the adhesive film from the Primer Plate to avoid contamination between wells.
14. Transfer 5 µL using reverse pipetting from each well in the Primer Plate to the inlets on the left side of the chip. Change pipette tips after each primer.
15. Transfer 5 µL of each sample using reverse pipetting from each well in position 1 A-H (green) to the inlets in the first column on the right side of the chip (green). Change pipette tips after each sample. When using an eight-channel pipette every other inlet will be filled according to the image.

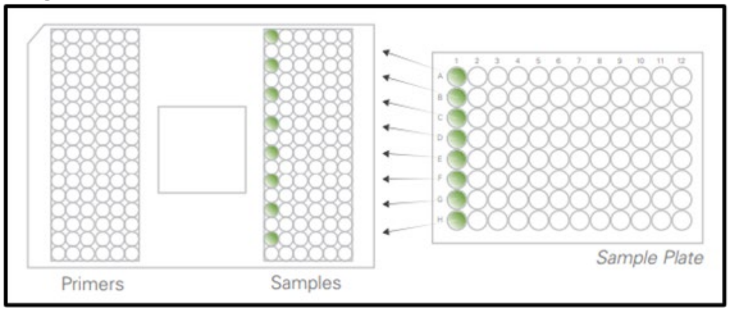

16. Transfer 5 µL from each well in position 2 A-H (blue) to the second column of inlets (blue) according to the image. Continue with columns 3-6. Use reverse pipetting and change tips between each column.

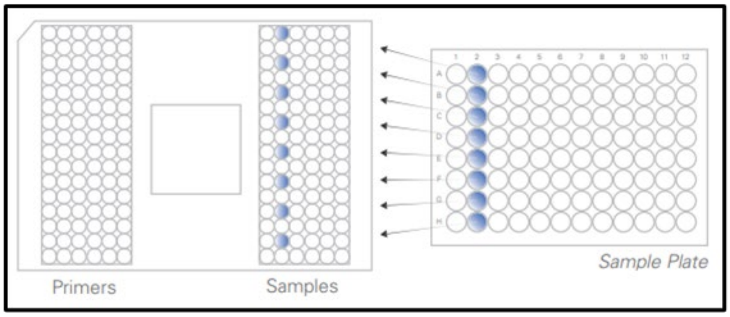

17. Transfer 5 µL from each well in position 7 A-H (red) to the inlets in the first column on the right side of the chip (red), start on the second row according to image. Continue with columns 8-12. Use reverse pipetting and change tips between each column.

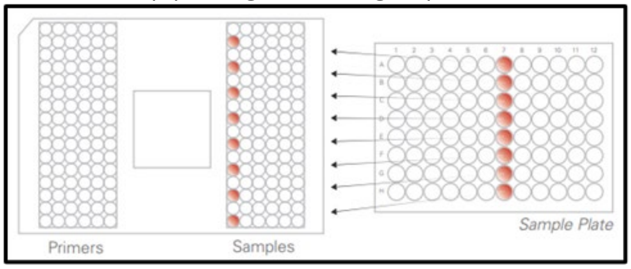

18. When loading is finished, inspect the wells and remove any bubbles using a syringe needle. Change the needle between wells to avoid contamination.
19. Use a piece of adhesive tape to remove dust from the top of the middle section of the IFC. Let the sticky part lightly touch the surface of the IFC.
20. On the screen on *Olink* Signature Q100, tap Open drawer. Place the correct interface plate on the IFC, put the IFC with the interface plate in the open drawer of the *Olink* Signature Q100. Make sure that the Interface plate is placed properly on the chip and in level with the drawer. Align the notched corner of the chip with the notch on the drawer and face the barcoded edges of the IFC and interface plate forward. Tap Close Drawer. On the Add run details screen, confirm the Chip ID, then enter either Run Name or Run Notes. Tap Start Run. The screen shows the remaining time.
21. When the run is complete, remove the IFC and interface plate from the instrument.

9.1.3 Antigen Array Assay (Mayo)

This protocol is adapted from Sasson et. al, Diverse Humoral Immune Responses in Younger and Adult COVID-19 patients mBio doi: 10.1128/mBio.01229-21.

Note: The first-generation multi-coronavirus protein microarray, produced by *ADI* (Irvine, CA, USA), will include full-length coronavirus structural and non-structural proteins from SARS-CoV-2 (WA-1), SARS-CoV, MERS-CoV, HCoV-NL63, and HCoV-OC43.

Procedure:

1. Purified proteins (baculovirus or mammalian cell culture produced) will be obtained from BEI Resources or expressed using an *E. coli* in vitro transcription and translation (IVTT) system (rapid translation system; *Biotechrabbit*, Berlin, Germany) and printed onto nitrocellulose-coated glass

Avid slides (*Grace Bio-Labs*, Inc., Bend, OR, USA) using an *Omni Grid* Accent robotic microarray printer (*Digilabs*, Inc., Marlborough, MA, USA).

2. Microarrays will be probed with sera (diluted 1:100 in PBS+ 2% albumin), and antibody binding will be detected by incubation with fluorochrome-conjugated goat anti-human IgG, IgA, or IgM (*Jackson ImmunoResearch*, West Grove, PA, USA, or *Bethyl Laboratories*, Inc., Montgomery, TX, USA).
3. Slides will be scanned on a *GenePix* 4300A high-resolution microarray scanner (*Molecular Devices*, Sunnyvale, CA, USA), and raw spot and local background fluorescence intensities, spot annotations, and sample phenotypes will be imported and merged in R. Downstream analyses will also be conducted in *R*.
4. Foreground spot intensities will be adjusted by subtraction of local background, and negative values will be converted to a value of 1.
5. All foreground values will be transformed using the base 2 logarithm. The data set will be normalized to remove systematic effects by subtracting the median signal intensity of the in vitro transcription and translation controls for each sample. With the normalized data, a value of 0.0 means that the intensity is no different than the background, and a value of 1.0 indicates doubling with respect to the background. For full-length purified recombinant proteins and peptide libraries, the raw signal intensity data will be transformed using the base 2 logarithm for analysis.

9.1.4 Hormone Analyses by mass spectrometry in Serum/plasma (OPBG)

Procedure: for Sample preparation with Steroid 96 SPE Well Plate
Equilibration of the Steroid 96 SPE Well Plate

1. Put Steroid 96 SPE Well Plate onto Waste Plate
2. Pipette 0.8 ml Equilibration Reagent 1 into each well of the Steroid 96 SPE Well Plate
3. Centrifuge 1 min at 400 x g, check for complete run through, discord effluent
4. Repeat steps 2 and 3 with 0.8 ml Equilibration Reagent 2

Sample preparation with Steroid 96 SPE Well Plate

1. Pipette 500 µl of the well homogenised sample/calibrator/control into each well of the Steroid 96 SPE Well Plate
2. Add 50 µl Internal Standard Mix and 450 µl Extraction Buffer
3. Shake Steroid 96 SPE Well Plate for 2 min at 600 rpm
4. Centrifuge 1 min at 400 x g, check for complete run through, discord effluent
5. Add 0.7 ml Wash Buffer and centrifuge 1 min at 400 x g, discord effluent
6. Add 0.7 ml Wash Buffer and centrifuge 2 min at 2000 x g to 3000 x g to dryness, discord effluent
7. Place Steroid 96 SPE Well Plate onto Steroid Collection Plate
8. Add 500 µl Elution Buffer to each well and centrifuge 1 min at 400 x g

Concentration and injection

1. Evaporate eluates in the Steroid Collection Plate under nitrogen or compressed air at 50 ° C to dryness
2. Reconstitute with 100 µl Reconstitution Buffer each in the Steroid Collection Plate
3. Shake Steroid Collection Plate for 2 min at 900 rpm

4. Seal Steroid Collection Plate with adhesive seal, transfer to autosampler
5. Inject 5-50 µl of each eluate into the LC-MS/MS system

9.2 PBMC processing

Assays planned for serum samples: Blood was obtained and PBMC will be isolated depending on the sites' protocol.

PBMC assays visual summary

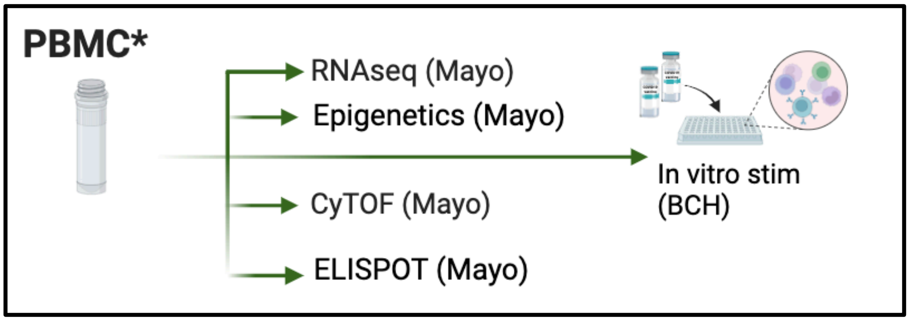

9.2.1 PBMC bulk RNA sequencing (Mayo)

RNA Extraction

1. Bring sample to room temperature.
2. Centrifuge the mix of cells and RNAprotect Cell Reagent (in Eppendorf tubes) for 10 min at 5000 x g with the hinge of the tube pointing outward. The pellet will be at the bottom and on the side under the hinge and it may be hardly visible.
3. Carefully, remove the supernatant by aspiration (use Pasteur pipette with the tip UP on the opposite side of the hinge, draw up the liquid by gradually tilting the tube). Loosen the pellet by flicking the tube.
4. Add 350 ul of Buffer RLT Plus (with 10 ul of b-ME/ per 1 mL RLT prepared before use). Vortex for 1 min.
5. Transfer the lysate into **QIAshredder spin column** and centrifuge for 2 min at full speed. Keep the flow-though (lysate)
6. Transfer the lysate to a **gDNA Eliminator spin column**. Centrifuge for 30 s at 13,300 rpm (17,000 x g). Keep the flow-though.
7. Discard the column and add 350 ul of 70% ethanol (prepared before use) in each collection tube and mix well by pipetting.
8. Transfer the 700 ul of each sample to a **RNeasy spin column** placed in a 2ml collection tube.
9. Centrifuge for 15 s at 13,300 rpm (17,000 x g). Discard the flow-through. At this point total RNA is bound to the membrane of the RNeasy spin column. Reuse the same collection tube.
10. Add 700 ul of Buffer RW1 to the RNeasy spin column.
11. Centrifuge for 15 s at 13,300 rpm (17,000 x g). Discard the flow-through. Reuse the same collection tube.
12. Add 500 ul of Buffer RPE to the RNeasy spin column.
13. Centrifuge for 15 s at 13,300 rpm (17,000 x g). Discard the flow-through. Reuse the same collection tube.
14. Add 500 ul of RPE to the RNeasy spin column. Centrifuge for 2 min at 13,300 rpm (17,000 x g).

15. Discard collection tube. Place column on **new 2 ml collection tube** and centrifuge at 13,300 rpm (17,000 x g) for 1 min.
16. Place the RNeasy spin column on **new 1.5 ml collection tube**.
17. Add 40 ul of RNase-free water directly on the spin column, wait 5 minutes, and centrifuge at 13,300 rpm (17,000 x g) for 1 min.
18. Pool RNA from same samples (if more than one tube/column) and place the RNA tube on ice. Measure the RNA concentration and record it.
19. Record the required information. Keep the checklist in the assay folder.
20. Store the RNA samples in -80 freezer. Remember to record also the samples exact location in the order as per the randomization list.

    NOTE: Samples in RNAprotect have to be stored at 4° C. They should be extracted within 4 weeks of the cell harvest. One typical batch for extraction includes 24 samples.

9.2.2 CyTOF (Mayo)
PBMC Staining

1. A vial of cryopreserved PBMC was thawed and washed.
2. The viability and cell count will be determined, and the cells will be washed in Cell Staining Buffer (**CSB**).
3. After the wash, the cells will be resuspended in CSB to a concentration of 6 × 107 cells/ml. FC receptors will be blocked by adding 5 µl of *Human TruStain FcX* to 3 × 106 cells in 50 µl and incubated for 10 min.
4. About 215 µl of CSB was then added to the PBMC.
5. About 270 µl of the PBMC was added directly to each of the four dry antibody tubes for antibody staining.
6. After a 30-min incubation, the cells will be washed twice in CSB, followed by fixation in 1.6% paraformaldehyde for 10 min.
7. Following fixation, the cells will be spun to a pellet, the fixative was removed, and the pellet was resuspended in 1 ml of the 125 nM *Cell-ID Intercalator-Ir* and incubated overnight at 4° C.

CyTOF Whole Blood Staining (Mayo)

1. An additional heparin blocking step was performed (100 U/ml) for 20 min at room temperature to reduce nonspecific binding between metal-tagged antibodies and eosinophils (Rahman, Tordesillas, & Berin, 2016).
2. Afterward, 270 µl of blood was added directly to four dry antibody tubes and allowed to incubate for 30 min at room temperature.
3. Immediately following staining, erythrocytes will be lysed by the addition of 250 µl of Cal-Lyse directly to the staining tube.
4. The tubes will be gently vortexed and allowed to incubate for 10 min at room temperature followed by the addition of 3 ml of *Maxpar* water and an additional 10 min of incubation.
5. The tubes are washed three times in *Maxpar* CSB followed by fixation in 1.6% paraformaldehyde for 10 min.
6. Following fixation, the cells are spun to a pellet, the fixative removed, and the pellet was resuspended in 1 ml of the 125 nm *Cell-ID*™ Intercalator-Ir (Ornatsky et al., 2008) and incubated overnight at 4° C.

CyTOF Sample Acquisition (Mayo)

1. Following the overnight incubation, the PBMC fixed cells are washed twice in CSB and twice with *Maxpar* Cell Acquisition Solution (**CAS**) with a final resuspension of the cells at 1 × 106 cells/ml in CAS containing 0.1× EQ™ Four Element Calibration Beads.
2. Whole blood sample acquisition was also performed the next day post staining on a Helios system utilizing *CyTOF®* Software version 6.7.1016 using the *Maxpar* Direct Immune Profiling Assay template. All instruments are equipped with a WB Injector, and all samples are acquired in CAS containing 0.1× EQ beads. All instruments will be evaluated to ensure performance at above the minimum Helios system specifications for calibration.
3. Following the instrument tuning and bead sensitivity test, the system was preconditioned with CAS. A minimum of 400,000 events for whole blood and 300,000 events for PBMC are acquired per file at a typical acquisition rate of 250–500 events/s.

9.2.3 In vitro stimulation assay (PVP)

In vitro assay preparation

1. Place 5 mL cold fetal bovine serum (**FBS**) in a 50 mL conical tube on ice in a BSL-2 biosafety cabinet.
2. Thaw vial of cryopreserved cells on ice until only a small clump of ice remains.
3. Transfer PBMCs to the pre-chilled, FBS-containing tube slowly, dropwise. Also dropwise, fill the tube to 50 mL with RPMI 1640 media
4. Centrifuge 10 minutes, 500 x g 4C.
5. After centrifugation, remove liquid from the cell pellet, add 1 mL RPMI 1640 containing 10% PPP and count.
6. Adjust volume with additional RPMI 1640/10%PPP to achieve a final concentration of 1.1 Million PBMCs per mL.

PBMC In vitro stimulation

1. Prepare all stimuli at 10X the desired concentration and plate 5 µL into a sterile, pyrogen-free, tissue culture treated round-bottom 96-well plate. Stimulations will be selected based on type of vaccine received by the participant (mRNA or Adenovirus), selecting synthetic PRR agonists targeting receptors hypothesized to be activated by the respective vaccines, see Table 7.
2. Plate 45 µL of cell suspension in RPMI/10% autologous platelet poor plasma (**PPP)** on top of each well. Incubate the plate in a 5% CO2 humidified incubator for 18-24 hours.
3. After incubation, remove 2 aliquots of 20 µL supernatant. One will be used for cytokine multiplexing/*Olink (*Beige label, see table 6*)*, the other can be used for proteomics and metabolomics.
4. Prepare Live/Dead stain by adding 1 µL *LIVE/DEAD*™ Fixable Blue Dead Cell Stain per 1 mL pure PBS (1:1000 dilution). Resuspend cells in a 50 µl diluted *LIVE/DEAD*™ stain and incubate at 4° in the dark for 30 min on a rocker.
5. Wash with 150 µL FACS buffer (PBS + 1% FBS), centrifuge at 400 x g for 5 min, and aspirate the supernatant.
6. Resuspend the pellet in minimum volume (approx. 10 µL of left-over volume), add 20 µL of Fc-block Hu FcR Binding Inhibitor solution (3 mL Fc Block + 17 mL FACS buffer), and incubate at 4° in the dark for 10 min. Note: Fc-block blocks the immunoglobulin Fc-receptors.

7. Add cell surface staining cocktail for each panel, diluting the antibodies with FACS buffer as shown in Table 4. Add 70 µL staining cocktail per well, gentle mix, and incubate at 4° in the dark for 30 min on the rocker.
8. Wash with 100 µL FACS buffer, centrifuge at 400 x g for 5 min, and aspirate the supernatant.
9. After a final wash of cell surface staining, agitate the plate by gently tapping to disrupt cell pellets (Only gentle tapping of the plate is required).
10. Add 100 µL of *Cytofix/Cytoperm* Buffer to each well. Incubate the plate for 25 minutes at 4oC in the dark. This step will fix the cell morphology and permeabilize the cells for subsequent intracellular staining.
11. Add 100 µL of 1× BD Perm/Wash buffer to each well and centrifuge the plate at 500 x g for 6 minutes. Aspirate the supernatant and repeat the washing step with 200 µL of 1× BD Perm/Wash buffer once.
12. Aspirate the supernatant from each well and agitate plate to disrupt cell pellets.
13. Add 80 µL desired intracellular staining cocktail. Each well should now have a total volume of 100 µl.
14. Incubate the 96-well plate for 45 minutes at 4oC in the dark on a rocker.
15. Add 100 µL of BD Perm/Wash buffer to each well and centrifuge the plate at 500 x g for 6 minutes at 4°C.
16. Aspirate the supernatant and agitate the plate to disrupt cell pellets.
17. Repeat the washing step. Add 200 µL of BD Perm/Wash Buffer to each sample well and centrifuge the plate at 500 x g for 6 minutes at 4°C. Aspirate the supernatant and agitate the plate to disrupt cell pellets. Resuspend cells in a final volume of 35 uL FACS buffer.
18. The samples are now ready for data acquisition and analysis on a flow cytometer.

Table 7. Stimuli for *in vitro* modeling. Pattern Recognition receptor (e.g. Toll-like receptor) agonists are purchased from *InvivoGen* (San Diego, CA; USA) except for MPLA purchased from *Avanti Polar Lipids* (Alabaster, AL; USA).

Priority Order for mRNA vaccine - Myocarditis		PRR Activated
1	Vehicle: PBS	non
2	Low dose (0.3 ug) mRNA vaccine (0.1 ug/uL)	TLR 7/8, MDA5, RIG-I, inflammasome
3	High dose (3 ug) mRNA vaccine (0.1 ug/uL)	TLR 7/8, MDA5, RIG-I, inflammasome
4	50 uM R848 (Invivogen)	TLR 7/8 + inflammasome
5	100 ng/mL Poly(I:C)-LMW/LyoVec™ (Invivogen)	RIG-I & MDA5
6	100 ng/mL High molecular weight Poly(I:C) (Invivogen)	TLR 3
7	1 ug/mL 3p-hpRNA (Invivogen)	RIG-I
8	Blank LNPs?	

Priority Order for adenovirus vaccine - TTS		PRR Activated
1	Vehicle: PBS	non
2	Low dose adenovirus vaccine (Janssen/AZ) (1:100 v:v)	TLR2,4,9 (some IFN are TLR-independent)
3	High dose adenovirus vaccine (Janssen/ AZ) (1:10 v:v)	TLR2,4,9 (some IFN are TLR-independent)
4	50 uM R848 (Invivogen)	TLR 7/8 + inflammasome
5	100 ng/mL Synthetic triacylated lipopeptide Pam3CSK4 (Invivogen)	TLR 2
6	1 ug/mL 3D-6A-PHAD MPLA (Avanti Polar Lipids)	TLR 4
7	5 uM CpG ODN 1018 (Invivogen)	TLR 9
8	50 ug/mL 2,3-cGAMP (Invivogen)	STING

9.3 RNA Sample Processing

<u>RNA assay visual summary</u>

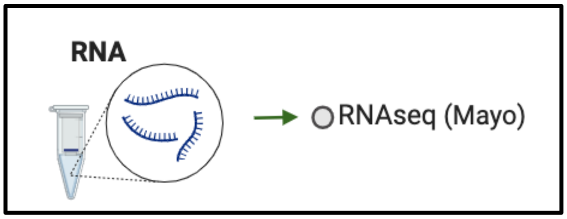

RNA Processing Procedures
Total RNA (including micro and small RNA) extraction is performed using the *Norgen* Preserved Blood RNA Purification Kit I as described in the kit manual. Follow the instructions below, using the indicated volumes, and refer to the product insert for extra details if required.

Working with RNA
RNases are very stable and robust enzymes that degrade RNA. Autoclaving solutions and glassware is not always sufficient to actively remove these enzymes. The first step when preparing to work with RNA is to create an RNase-free environment. The following precautions are recommended as your best defense against these enzymes.

- The RNA area should be located away from microbiological workstations.
- Clean, disposable gloves should be worn at all times when handling reagents, samples, pipettes, disposable tubes, etc. It is recommended that gloves are changed frequently to avoid contamination.
- There should be designated solutions, tips, tubes, lab coats, pipettes, etc. for RNA only
- All RNA solutions should be prepared using at least 0.05% DEPC-treated autoclaved water or molecular biology grade nuclease-free water.
- Clean all surfaces with commercially available RNase decontamination solutions.
- When working with purified RNA samples, ensure that they remain on ice during downstream applications.
- All centrifugation steps are carried out in a benchtop microcentrifuge at 14,000 x g except where noted. All centrifugation steps are performed at room temperature.
- A variable speed centrifuge should be used for maximum kit performance. If a variable speed centrifuge is not available a fixed speed centrifuge can be used, however reduced yields may be observed.
- Ensure that all solutions are at room temperature prior to use.
- Prepare a working concentration of the Wash Solution by adding 50 mL of 95% ethanol (provided by the user) to the supplied bottle containing the concentrated **Wash Solution**. This will give a final volume of 72 ml. The label on the bottle has a box that may be checked to indicate that the ethanol has been added.
- It is important to work quickly during this procedure.

Lysate Preparation from Tempus™ Blood RNA Tubes
If processing frozen *Tempus™* blood tubes, allow the blood to thaw completely in the tube at room temperature, inverting the tube occasionally (~30-40min).

1. Pour the entire contents of the *Tempus™* tube into a new 50 mL conical tube.
2. Add 3 mL of *Tempus™* Blood RNA Tube Diluent (or enough to adjust the final volume to 12 mL).
3. Close the tube tightly and mix by vortexing vigorously for 30 seconds.

4. Centrifuge the tube at 4°C at 3000 – 5000 x g (minimum 4500 rpm) on a Beckman JB-6 or equivalent swing bucket centrifuge for 30 minutes.
5. Carefully discard supernatant. *Note: The RNA pellet is transparent and invisible.*
6. Place the inverted tube on absorbent paper for 1-2 minutes, then blot any remaining drops from the rim with clean absorbent paper.
7. Add 600 µl of Lysis Solution to the RNA pellet. Vortex briefly to resuspend the pellet.
8. Add 300 µL of 95-100% ethanol (provided by the user). Vortex briefly to mix.
9. Keep resuspended RNA pellet on ice while preparing for the next steps.

Binding RNA to column

1. Assemble a column with one of the provided collection tubes
2. Apply up to 600 µL of the lysate with the ethanol mix onto the column and centrifuge for 1 minute at ≥ 3,500 x g
   Note: Ensure the entire lysate volume has passed through into the collection tube by inspecting the column. If the entire lysate volume has not passed, spin for an additional minute at 14,000 x g
3. Discard the flowthrough. Reassemble the spin column with its collection tube.
4. Repeat Step 2 and 3 as necessary.

Column Wash

1. Apply 400 µL of Wash Solution to the column and centrifuge for 1 minute at 14,000 x g
   Note*:* Ensure the entire wash solution has passed through into the collection tube by inspecting the column. If the entire wash volume has not passed, spin for an additional minute.
2. Discard the flowthrough and reassemble the spin column with its collection tube.
3. Repeat steps 1 and 2 for 2 additional times (a total of 3 washes)
4. After the last spin, spin the column for an additional 2 minutes at 14,000 x g to thoroughly dry the resin. Discard the collection tube.

RNA Elution

1. Place the column into a fresh 1.7 mL Elution tube provided with the kit.
2. Add 50 µL of Elution Solution to the column.
3. Centrifuge for 2 minutes at 200 x *g*, followed by 1 minute at 14,000 x *g*
   Note: The volume eluted from the column. If the entire 50 µL has not been eluted, spin the column at 14,000 x g for 1 additional minute.
   Note: For maximum RNA recovery, it is recommended that a second elution be performed into a separate microcentrifuge tube (Repeat Steps 2 and 3).

Storage of RNA

The purified RNA sample may be stored at –20°C for a few days. It is recommended that samples be placed at a minimum of –80°C for longer term storage (up to 2 years) but at –150°C or in liquid nitrogen for long term storage.

9.4 Assays planned for plasma samples

Plasma was obtained from blood using site-specific protocols.

Plasma assays visual summary

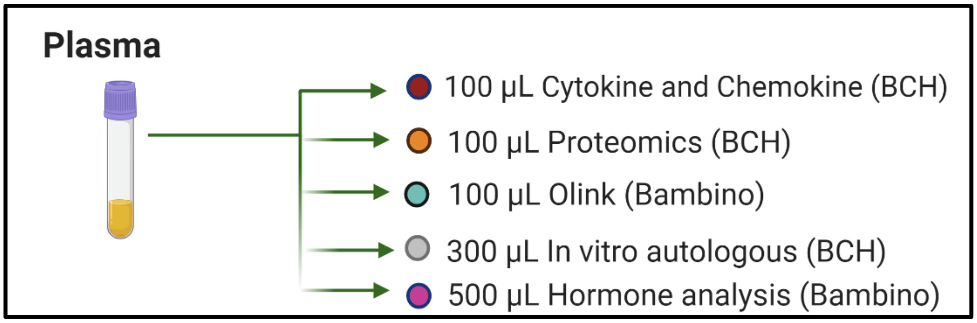

9.4.1 Mass-spectrometry-based discovery proteomics (BCH-Steen Lab)

Goal: Process plasma or serum for a comprehensive mapping of the 'classical' and 'tissue leakage' plasma proteome (REF: PMID12488461) for comprehensive plasma protein-based immunophenotyping. To ensure optimal analytical depth, a 2-pronged approach is used – one for the 'classical' plasma proteome and one for the 'tissue leakage' plasma proteome.

Method 1: 'Classical' plasma proteome mapping using neat plasma

Purpose: High throughput processing of plasma and/or serum samples for the mapping of the "classical plasma proteome".

Materials:

- Tabletop *Eppendorf* Centrifuge
- *Eppendorf* tubes, 1.5 mL
- Magnetic rack
- Thermomixer
- Absolute Ethanol
- Acetonitrile (LC/MS)
- Water (LC/MS)
- Formic acid (LC/MS)
- Urea (cat. No. 51456-2.5KG)
- Ammonium bicarbonate (cat. No. 09830-1KG)
- *Sera-Mag SpeedBeads* (*GE Healthcare,* cat. No. 45152105050250; 1 µm avg. part. size, suspension (5% Solids)) stock 50 mg/ml
- *Sera-Mag SpeedBeads* (*GE Healthcare,* cat. No. 65152105050250; 0.70-1.10 µm avg. part. size, suspension (5% Solids)) stock 50 mg/ml
- Iodoacetamide (**IAA**) (cat. No. I1149-25G)
- Dithiothreitol (**DTT**) (cat. No. D9779-25G)
- Trypsin Gold 100 µg (*Promega*, V5280)
- Formic acid (**FA**)

Reagents: Prep of buffers:

- 50 mM Ammonium bicarbonate (**ABC**): 4 g in 1 L water
- 8M Urea buffer: 242 g of urea + 300 mL 50 mM Ammonium bicarbonate (**ABC**)
- 0.05 M DTT (m = 74 mg in 10 ml 8 M Urea buffer)
- 0.375 M IAA (m = 690 mg in 10 ml 8 M Urea buffer)
- Trypsin: resuspend each trypsin 100 µg vial in 4 mL 50 mM Ammonium bicarbonate

Step-by-Step Protocol

Step 1: Prepare bead mixture

Per sample (50 µg protein, 1 µL volume):

1. Add 10 µL of each of the two *SpeedBeads* to a microcentrifuge tube and place on the magnetic rack, wait until beads collect on side of tube, remove supernatant.
2. Wash with 2X with 200 µL water.
3. Resuspend in 10 µL water.

Step 2: Sample processing / Digestion

4. Combine the following:
    - 8 M Urea buffer (in 50 mM ABC) => Volume = 60 µL.
    - Plasma/Serum sample => Volume = 1 µL.
    - 0.05 M DTT (m = 7.4 mg in 8 M Urea buffer) => Volume = 15 µL.
5. Incubate for 30 minutes at RT with shaking at 1000 rpm
6. Add 5 µL of 0.375 M IAA (m = 69 mg in 8M Urea buffer). Incubate for 30 minutes at RT in dark.
7. Add 10 µL of 0.05 M DTT. Incubate 15 minutes on bench to quench alkylation.
8. Transfer the sample buffer (Volume ~ 91 µL) in SP3 bead mixture (scale 1:10 protein to beads).
9. Add 150 µL absolute ethanol, incubate 10 minutes at RT.
10. Place on magnetic rack and wait until supernatant is clear, remove supernatant.
11. Wash 3X with 200 µL 80% ethanol by placing on magnetic rack, removing supernatant, and resuspending in wash solution by pipetting up and down 4 times.
12. Place in magnetic rack, remove supernatant, and add 100 µL 50 mM ABC containing 2 µg trypsin (1: 25 trypsin to proteins) (V = 50 µl from 100 µg trypsin reconstituted in 2 mL 50 mM ABC).
13. Resuspend the beads by pipetting them up and down.
14. Incubate for 2 hours at 1000 RPM at 37 °C on thermomixer.
15. Centrifuge 1 min at 20000g (10 min at 3220 g).
16. Place on magnetic rack and remove supernatant, discards beads.
17. Acidify supernatant by adding 2 µL FA (2% v/v) to quench digestion.

Step 3: Loading of *Evotip* for subsequent LC/MS analysis

18. Evotips are washed with 20 uL 0.1% formic acid in acetonitrile through centrifugation at 700 ×g for 60 s.
19. Evotips are soaked in 1-propanol for 1 min until pale white
20. Evotips are washed with 20 uL 0.1% formic acid in water at 700 ×g for 60 s.
21. 5 uL of the SP3 digest (i.e., ~200ng of digest) are diluted into a final 20 uL volume with 0.1% formic acid in water.

22. 20 uL of the diluted digest is loaded onto Evotips through centrifugation at 700 ×g for 60 s.
23. Another 20 uL volume of 0.1% formic acid in water is centrifuged through to wash the Evotips.
24. 200 uL of 0.1% formic acid in water is added onto the Evotips, which is centrifuged for 10 s at 700 ×g.

Method 2: 'Tissue leakage' plasma proteome mapping based on the biochemical depletion of the most abundant plasma proteins.
Purpose: Depletion of abundant proteins to detect lesser abundant proteins

Equipment/Supplies/Reagents:

- Experiments is done in 96 deep-well plates 0.8 mL
- µSPE Hydrophilic-Lipophilic-Balanced (**HLB**) plate prep (W*aters* cat# 186001828BA)
- Extraction Plate Manifold for *Oasis* 96-Well Plates (*SKU*: 186001831) https://www.waters.com/nextgen/us/en/shop/sample-preparation--filtration/186001831-extraction-plate-manifold-for-oasis-96-well-plates.html
- Tabletop *Eppendorf* Centrifuge 5810R
- Methanol (LC/MS)
- Acetonitrile (LC/MS)
- Water (LC/MS)
- Formic acid (LC/MS)
- Trifluoracetic acid (**TFA**, LC/MS)

Reagents: Prep of buffers:

- 70 % perchloric acid (*Sigma Aldrich* 311421-50ML)
- 1% trifluoroacetic acid (1 mL TFA in 100mL LC/MS grade water)
- 0.1% trifluoroacetic acid (1 mL TFA in 1000mL LC/MS grade water)
- 90 % acetonitrile 0.1% trifluoroacetic acid (90 mL LC/MS acetonitrile, 10 mL LC/MS water, 100 uL TFA)
- 10 % formic acid (1mL formic acid (LC/MS grade) + 9 mL water (LC/MS grade))

Step-by-Step Protocol:

Step 1. PerC Acid step

1. Add 450 µL of LC/MS water to 50 µL of plasma (shake ~ 1 min)
2. Add 25 µL of perchloric acid (70% i.e. straight from the bottle) (shake at 1200 rpm on a thermomixer ~ 3 min). It should get very cloudy and after shaking it should be homogeneous.
3. Place the plate at -20°C for 15 min
4. Centrifuge for 60 min (4C, 3200 g) (tabletop centrifuge, we use an *Eppendorf* Centrifuge 5810R)

Step 2. Preparation of the HLB plate and sample cleanup

Preparation of HLB plate

5. (During the plate centrifugation) conditioning of the µSPE HLB plate using the plate manifold for *Oasis* 96-Well Plates:
6. Add 300 µL methanol (LC/MS grade), aspirate (apply vacuum)

7. Add 500 µL of 0.1% trifluoroacetic acid, aspirate
8. Add 500 µL of 0.1% trifluoroacetic acid, aspirate
9. The plate is now conditioned and ready for sample loading
6. Add 40 µL of 1% trifluoroacetic acid in a new plate (Plate 2)
7. After centrifugation transfer 390 µL of the supernatant (it is crucial to not pipette the pellet) from plate 1 into plate 2 (using a liquid handling robot such as the *Opentrons OT-2*; or multi-channel pipette)
8. Transfer plate 2 onto the µSPE HLB (*Waters* 186001828BA) plate (which was previously conditioned, sample loading step).
    - Apply vacuum, aspirate slowly at -5 in.Hg (~ -120 mm.Hg)
9. Wash 1x with 500 µL of 0.1% trifluoroacetic acid
    - Apply vacuum, aspirate slowly at -5 in.Hg (~ -120 mm.Hg)
10. Elute with 100 µL 90 % acetonitrile 0.1% trifluoroacetic acid in a new plate (Plate 3)
11. Dry in *Speedvac* (until dry. It will take 1-2 h. If there is a brownish pellet at the bottom of the well it means that some perchloric acid is left and the sample has to be discarded.)

Step 3. Sample resuspension

12. The dry samples will be resuspended with 40uL of 50 mM ammonium bicarbonate and digested by adding 5uL trypsin (5 uL here correspond to 500 ng, *Promega*) 18h at 37C. Digestion is stopped by the addition of 5 uL 10% formic acid. Tryptic peptides are kept at -80C until LC/MS analysis.

Step 4. Loading of *Evotip* for subsequent LC/MS analysis

13. Evotips are washed with 20 uL 0.1% formic acid in acetonitrile through centrifugation at 700 ×g for 60 s.
14. Evotips are soaked in 1-propanol for 1 min until pale white
15. Evotips are washed with 20 uL 0.1% formic acid in water at 700 × g for 60 s.
16. 5 uL of the digest after the perCA precipitation (i.e., ~200ng of digest) are diluted into a final 20 uL volume with 0.1% formic acid in water.
17. 20 uL of the diluted digest is loaded onto Evotips through centrifugation at 700 ×g for 60 s.
18. Another 20 uL volume of 0.1% formic acid in water is centrifuged through to wash the Evotips.
19. 200 uL of 0.1% formic acid in water is added onto the Evotips, which is centrifuged for 10 s at 700 × g.

Note: both protocols are agnostic to the type of anti-coagulant used for plasma preparation. Furthermore, they can also be used for serum.

9.4.2 Soluble Cytokines and Chemokines Quantification assay (PVP):

Goal: Quantify the cytokines and chemokines released in the plasma using a custom-made multiplex kit

Equipment/Supplies/Reagents:

- Human cytokine/chemokine multiplexing kit (*EMD-Millipore*# HCYTOMAG-60K-41)
- *Corning Cellbind* 384-well plates (*Sigma*# CLS-3683-50EA)
- Assay diluent: 5X provided in kit, dilute using diH2O

- Wash buffer: 20X provided in kit, dilute using diH2O
- Handheld Magnetic Separator Block (*EMD-Millipore*# 40-285)
- D-PBS (*Gibco* # 14190-250)
- *Flexmap 3D* multiplexing analyzer (*Luminex*)
- Flexmap Calibration/verification kit *(Luminex*)
- *Luminex xponent version 4.2 software*
- *Milliplex analyst software*

Procedure:

Day 1:

Samples have been frozen at -80°C and/or shipped on dry ice. Take samples out of the freezer and place on wet ice.

1. Thaw the sample between 4°C-15°C for 15 min. Check that sample has completely thawed by inverting, ensuring all of the solution is liquid again.
2. Centrifuge the samples at 600g for 5mins.
3. Open the multiplexing kit. Prepare standard curve and quality control vials as instructed in the kit manual.
4. Bead preparation is slightly different from the manual. Prepare the beads as following:
    a. If beads are already pre-mixed, add assay diluent to the beads, so that the final volume is 4.5 ml.
    b. Sonicate for 30 seconds.
    c. If the beads came separately, sonicate each vial for 30 seconds. Check that all different cytokines (bead regions) are present in the kit. Vortex the first bead vial for 30 seconds and pipet 85 µl into the bead mixing bottle provided. Repeat for all beads. Add assay diluent to the beads, so that the final volume is 4.5 ml.
5. In a new 384-well plate, pipet 20 µl of standards and blanks (duplicates) and QC1/QC2 (single replicates) into column 1. Add 20 µl of serum matrix (provided in kit) to all wells in column 1 for buffer matching with the experimental samples.
6. The remaining wells (352) can be used for samples. Samples are run in triplicates. That means each plate can accommodate 117 samples. It is recommended to make a plate layout ahead of time. Add 20 µl of sample to each designated well. Add 20 µl of assay diluent to each sample well for buffer matching with the standards/QCs.
7. To all wells in the plate, add 10 µl of the prepared bead mixture. Make sure to mix well and keep mixing intermittently throughout the dispensing process, to ensure equal bead distribution to the wells. Bead adding can be done with a multichannel pipette or repeat pipettor.
8. Seal the plate and incubate overnight at 4°C, shaking at 750 rpm.

Day 2:

1. Take the plate and plate shaker out of the cold room, allow it to reach room temperature before removing seal from the plate, to prevent condensation into the plate. Wash the plate using the following plate washing instructions:
    a. At room temperature, incubate the plate on a plate magnet for 2 minutes.

b. While holding the plate on the magnet, flick the plate empty into waste disposal.
c. Add 50µl wash buffer to each well.
d. Incubate magnet on plate again for 2 minutes
e. While holding the plate on the magnet, flick empty again
f. Add another 50 µl wash buffer
g. Leave plate on magnet for another 2 minutes
h. While holding the plate on the magnet, flick empty again
i. Leave plate on magnet for another 2 minutes
j. While holding the plate on the magnet, tap the plate dry on a paper towel
k. Add 6 µl of detection antibodies to each well. Incubate the plate at RT, 750rpm, 1 hour
l. DO NOT WASH THE PLATE

2. Add 6 µl of Strep-PE to each well. Incubate the plate at RT, 750rpm, 30 minutes
3. Wash the plate as described in step 2.10.
4. Add 35 µl of PBS to each well, shake at RT, 750 rpm for 5 minutes before loading plate in the *Flexmap.*

Note: Refer to the sections above, 9.1.2 for *OLINK* and 9.1.4 Hormone analysis

9.5 Saliva sample processing

Note*: While most the sites will be able to share saliva samples for genotyping, other sample types may be shared in the absence of saliva.*

<u>Saliva assay visual summary</u>

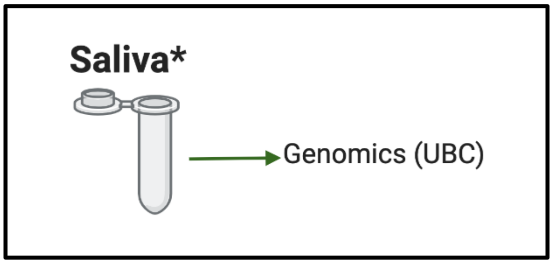

Saliva DNA sample shipment
Collected saliva samples can be stored and shipped at room temperature.

Saliva DNA storage
Oragene® collection kits are stable at room temperature for years because of proprietary reagents that prevent bacterial growth and minimize chemical hydrolysis of DNA. Saliva DNA samples will be stored as received in the secure locked area at the BCCHRI, and DNA will be extracted once samples are ready for genotyping and analyses.

Participants retain control of their DNA samples. If participants consent, their genetic material will be bio-banked and stored in a freezer in the Carleton Laboratory at BCCHRI to preserve it for future testing for an indefinite period. No personal identifying information will be labeled on the DNA sample tube.

DNA extraction

1. Prior to genomic DNA extraction, saliva samples will be incubated at 50°C for 2 hours.

2. Genomic DNA will be extracted and purified using established automated methods using the Qiagen *QiaSymphony®* high-throughput platform.
3. Extracted DNA will be eluted directly into a Matrix *ScrewTop®* tube in a 96 well Matrix plate (*Thermo Fisher Scientific*).
4. Each unique 2D barcode on the Matrix *ScrewTop®* tube will be entered into the Laboratory Information Management System (**LIMS**).
5. Genomic DNA samples will be then quantified using a *Quant-iT PicoGreen dsDNA Assay Kit®* (*Invitrogen*).
6. Extracted DNA samples will be stored immediately at -20°C and be moved to -80°C after completing genotyping for long-term, at least 4 years, storage.

9.5.1 Genotyping (UBC):

Genome-wide association studies (GWAS)

Extracted DNA from saliva samples will be genotyped with a custom *Illumina* Global Screening Array (GSA version 3.0 with additional pharmacogenomic content) including genetic variation throughout the genome (500,000 genome-wide markers), further enriched with pharmacogenomic variants including >45,000 variants in core drug absorption, distribution, metabolism, and excretion genes, and >24,000 variants in major histocompatibility complex (**MHC**)/HLA gene regions. The array captures both common and rare variations collected from large-scale sequencing projects.

Previously reported candidate genes potentially related to the pathogenesis or biological mechanisms of specific COVID-19 vaccine-induced GBS, VITT, and myocarditis/pericarditis will be also genotyped by either the custom GSA array we will be used or custom TaqMan genotyping assays. Candidate genes for GBS include, but not limited to, HLA alleles, IL-10, KIR, TNF-α, CD1, and FcγR. Candidate genes for VITT include, but are not limited to, F5, F2, PROC, and PROS1. Candidate genes for myocarditis/pericarditis include, but are not limited to, BAG3, DSP, PKP2, RYR2, SCN5A, and TNNI3.

Genotyping will be followed by (1) whole genome imputation of common variants using SHAPEIT (v2) and IMPUTE2 (v2.3.2) in combination with the Phase 3 1000 Genomes Project reference panel and (2) imputation of classical HLA alleles and HLA-region variants using SNP2HLA (v1.0.2) in combination with Type 1 Diabetes Genetics Consortium (T1DGC) reference panel. This will yield a final genotyped/imputed dataset of approximately 10 million variants per sample.

9.5.2 Exome sequencing (ES)

From each of the three 275 case groups, 50 of each the most severe AE patients who are categorized as Brighton Collaboration Level One cases of COVID-19-induced GBS, VITT, or myocarditis/pericarditis will also be selected (a total is 150 for three AEs) to perform ES analysis. This will complement genome-wide genotyping, particularly in protein-coding regions, to identify the most possible disease-causing mutations. The public ES database – *gnomAD* – as reference controls will be used to investigate novel and rare genetic variants related to these three specific AEs. Following library preparation with an *IDT* Capture Expanded Exome Kit, exome sequencing to a mean coverage of 100X will be performed using paired end sequencing (2 x 150 bp) on an *Illumina* Sequencing platform (*NovaSeq* platforms). The sequence data will be processed according to GATK Best Practices (v4), using BWA-MEM for alignment of reads to the GRCh38 reference genome on the local high-performance computing cluster.

Significant variants identified from the GWAS and ES discoveries will be further validated by genotyping (e.g., TaqMan assays) or sequencing.

****

END of Standard Operating Procedure
